# Broadband opto-thermal camouflage and infrared encrypted communication via inverse design

**DOI:** 10.1038/s41377-026-02370-x

**Published:** 2026-06-22

**Authors:** Qixiang Chen, Chengcong Li, Zhuning Wang, Zezhao Ju, Jieren Song, Hongtao Lin, Huajie Tang, Chengyue Guo, Yaoguang Ma, Xun Cao, Dongliang Zhao

**Affiliations:** 1 School of Energy and Environment, Southeast University, Nanjing, Jiangsu 210096 China; 2 State Key Laboratory of Functional Crystals and Devices, Shanghai Institute of Ceramics, Chinese Academy of Sciences, Shanghai, 201899 China; 3 Center of Materials Science and Optoelectronics Engineering, University of Chinese Academy of Sciences, Beijing, 100049 China; 4 State Key Laboratory for Extreme Photonics and Instrumentation, College of Optical Science and Engineering, Zhejiang University, Hangzhou, Zhejiang 310013 China; 5 The State Key Lab of Brain-Machine Intelligence, Key Laboratory of Micro-Nano Electronics and Smart System of Zhejiang Province, College of Information Science and Electronic Engineering, Zhejiang University, Hangzhou, Zhejiang 310027 China; 6 Institute of Science and Technology for Carbon Neutrality, Southeast University, Nanjing, Jiangsu 210096 China

**Keywords:** Photonic crystals, Metamaterials

## Abstract

Multispectral detection technologies spanning optical and thermal bands pose a severe threat to military camouflage while simultaneously unlocking new opportunities for covert communication. However, smart materials capable of both countering these threats and exploiting these bands for communication are still lacking. Here, using a Bayesian-optimization-based inverse-design strategy, we propose an opto-thermally decoupled photonic structure. It features broadband optical camouflage across the 0.38–2.5 μm range, encompassing the visible, near-infrared, and short-wave infrared bands (including the 1.55 μm laser wavelength), with a tunable structural-color palette that covers 66% of the CMYK gamut. Crucially, while these colors are independently tunable, the structure modulates radiance in the mid-wave infrared (MWIR) and long-wave infrared (LWIR) bands for dynamic thermal camouflage, achieving consistent MWIR/LWIR emissivity switching between 0.41 ± 0.04/0.90 ± 0.01 and 0.93 ± 0.03/0.45 ± 0.01 driven by the vanadium dioxide (VO₂) phase transition. Beyond camouflage, by precisely regulating the temperature, we exploit the differential MWIR/LWIR thermal signatures generated by the continuous phase evolution of VO₂ to encode information for infrared encrypted communication. We experimentally demonstrate the structure’s dual capabilities for broadband opto-thermal concealment and covert communication. This work integrates multispectral camouflage and covert communication within a single platform, offering a new design strategy for next-generation military smart materials.

## Abstract (web summary)

A photonic structure achieving broadband opto-thermal camouflage and infrared encrypted communication, advancing adaptive concealment and signaling.

## Graphical abstract

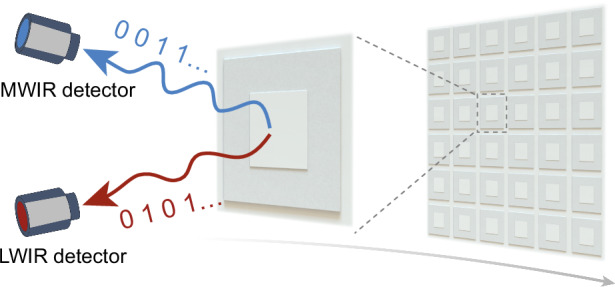

## Introduction

Advanced detector technologies can capture the optical and thermal signatures of targets across multiple spectral bands, posing a severe threat to the safety of military personnel and equipment, while presenting unprecedented challenges to military camouflage capabilities^1,2^. Camouflage aims to alter the observable signatures of a target so that it blends with the environment, thereby evading detection^3,4^. Crucially, the target’s observable signatures themselves present a unique opportunity for encrypted information transfer: using non-visible bands to encode and securely transmit information^5–10^. Modern military detection systems operate across several critical spectral bands. These include the reflected-light imaging bands of the visible (VIS, 0.38–0.78 μm), near-infrared (NIR, 0.78–1.4 μm), laser (hereafter referring to 1.55 μm), and short-wave infrared (SWIR, 1.4–2.5 μm), where sensors form optical images by capturing reflected solar or artificial illumination. In contrast, the thermal detection bands, encompassing the mid-wave infrared (MWIR, 3–5 μm) and long-wave infrared (LWIR, 8–14 μm), operate by detecting the thermal radiation emitted by the object itself. Confronted with detection technologies covering such a broad spectral range, the development of multispectral camouflage strategies is essential.

Since the early 20th century, pigments have been systematically used to create camouflage patterns to reduce the risk of exposure^3,4^. Pigments, which rely on the mechanism of photon-excited electron transitions for coloration, are effective only in the high-photon-energy VIS band^11,12^. When detection occurs outside the VIS band, the optical discrepancies between the target and the environment become evident. Eliminating this optical discrepancy requires an alternative coloring mechanism capable of generating differential light reflection across the broadband spectrum to achieve effective NIR and SWIR camouflage. Artificial structural color offers this possibility; it achieves coloration through the interaction of light with surface structures, and the high degree of freedom in structure design enables broadband optical camouflage structural colors^13–16^. On the other hand, the key to eliminating thermal signature discrepancies lies in controlling the emissivity. According to the Stefan-Boltzmann Law ($P=\varepsilon \sigma {T}^{4}$, where *σ* denotes the Stefan-Boltzmann constant, *ε* represents the emissivity, and *T* stands for the absolute temperature of the object), the radiant power of an object is directly proportional to its emissivity^17,18^. Therefore, for an object at a specific temperature, its thermal signature can be merged with the background by modifying its emissivity within the MWIR and LWIR bands, thereby reducing the risk of thermal exposure. Researchers have proposed various strategies, including those for the VIS^19–22^, thermal^2,23–27^, and combined VIS-thermal bands^28–33^. However, these methods often lack the spectral decoupling required to separate adjacent optical and thermal bands with small wavelength gaps—a prerequisite for genuine broadband opto-thermal camouflage. More importantly, the focus on unimodal or single-function camouflage forfeits the opportunity to exploit these bands for information exchange. This design challenge for smart materials spanning multiple spectral bands remains formidable.

In this work, employing a Bayesian optimization (BO)-based inverse-design methodology, we propose a photonic structure that seamlessly integrates broadband opto-thermal camouflage with infrared encrypted communication. The inverse-designed structure features highly customizable structural colors covering a wide color gamut, while ensuring broadband optical concealment across the VIS, NIR, and SWIR bands. In the thermal spectrum, the structure can selectively operate in either thermal camouflage or thermal communication mode as required. In specific thermal environments or when thermal communication is necessary, dynamic thermal camouflage or communication can be achieved by actively modulating the temperature, which alters the structure’s radiance in the MWIR and LWIR bands. This capability stems from the distinct thermal signatures generated in the MWIR/LWIR bands by the continuous phase evolution of vanadium dioxide (VO_2_). Importantly, the opto-thermal decoupling characteristic of the structure ensures that stable optical camouflage is maintained during the dynamic switching of thermal signatures. This work provides a strategy for camouflage in complex opto-thermal environments and a backup link for encrypted communication in complex electromagnetic environments, offering a viable route to tactical communication under extreme conditions.

## Results

### Principle of the photonic structure

**Fig. 1:**
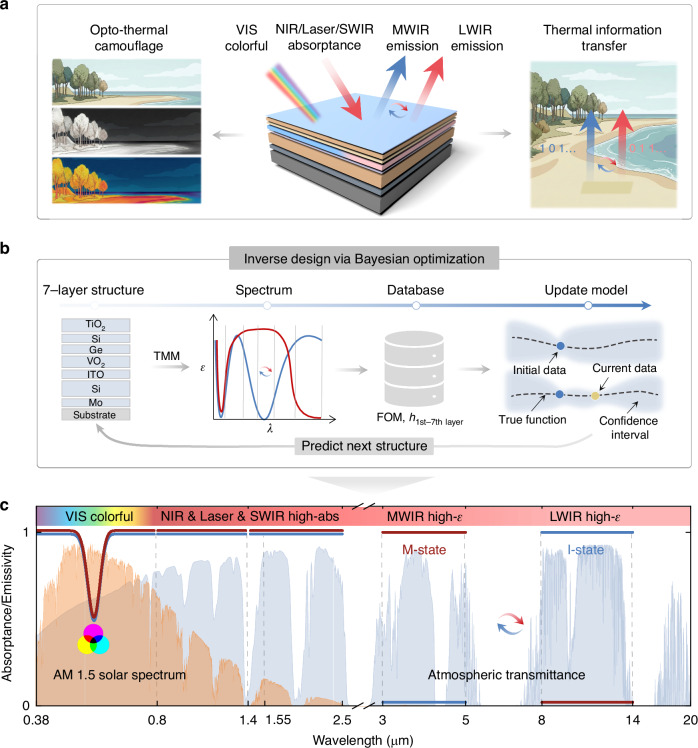
Schematic of the photonic structure and its inverse-design flow. **a** Schematic representation of the photonic structure (center), outlining its applicable camouflage scenarios and spectral functionalities (left), and its potential for encrypted information transfer via thermal radiation signature (right). **b** Schematic illustration of the BO-based inverse-design process. **c** The target spectrum of the photonic structure yielded by the BO-based inverse design

As illustrated in Fig. 1a, the proposed photonic structure is designed to simultaneously customize distinct camouflage colors in the VIS band according to the scene, maintain optical camouflage across the NIR, laser, and SWIR bands, and dynamically switch between selective high emissivities in the MWIR and LWIR bands. This comprehensive spectral control strategy facilitates day–night optical camouflage and selective thermal camouflage (Fig. 1a, left). To overcome the structural design challenges posed by multispectral customization and dynamic modulation, we used a BO-based inverse-design method to precisely tailor the complex multispectral response^34,35^. As shown in Fig. 1b, the initial photonic structure is configured as a seven-layer stack, from top to bottom: titanium dioxide (TiO_2_), top silicon (Si), germanium (Ge), VO_2_, indium tin oxide (ITO), bottom silicon (Si), and molybdenum (Mo). Spectral characteristics are calculated using the transfer matrix method (TMM) and quantified by a figure of merit (FOM). Subsequently, a surrogate function is constructed using the Gaussian process regression model, and an acquisition function guides the iterative sampling of layer thicknesses, predicting the optimal structural parameters until the FOM reaches the global minimum. The VO_2_ layer is pivotal for the dynamic MWIR/LWIR emissivity switching, as its insulator-to-metal transition produces a transparent-to-reflective shift in the mid-infrared regime. Therefore, the proposed seven-layer structure functions as a dual Fabry–Pérot (F-P) resonant cavity. In the insulator state (I-state), the structure operates as an F-P cavity where the bottom Si layer serves as the lossless dielectric spacer; after phase transition to the metal state (M-state), the TiO_2_ /top Si/Ge stack effectively becomes the lossless dielectric layer of the transformed F-P cavity. The target spectrum used to guide the inverse design (Fig. 1c) features freely customizable VIS reflectance (enabling diverse structural colors), high absorption across the NIR–SWIR range (including the 1.55 μm laser wavelength), and dynamically switchable selective high emissivity in the MWIR and LWIR atmospheric windows.

However, such selective emission and temperature-dependent switching are subject to inherent thermodynamic constraints. Under ambient conditions, active heating inevitably increases the radiance of the structure, making it more conspicuous to MWIR and LWIR detectors. Because thermal communication itself relies on the thermal signature produced by this active heating, the thermal-camouflage and thermal-communication functions are governed by a fundamental physical trade-off. We therefore define the operational framework as follows: in ambient conditions, the thermal-communication function remains “normally off” so that thermal contrast with the background is minimized and passive thermal stealth is preserved; active heating is engaged only when thermal communication is required, or when the surrounding thermal background is compatible with the heated state. This integrated scheme provides a backup link for encrypted communication in electromagnetically congested environments while preserving the option of thermal camouflage in specific thermal scenarios.

### Bayesian optimization-based inverse design

The FOM used in the BO-based inverse-design process is defined by the following equation:

$${\rm{FOM}}={{\rm{W}}}_{1}{(R}_{{\rm{NIR}}-{\rm{SWIR}}}\left({\rm{I}}\right)+{R}_{{\rm{NIR}}-{\rm{SWIR}}}\left({\rm{M}}\right)-2)+{{{\rm{W}}}_{2}(R}_{{\rm{MWIR}}}({\rm{M}})-{R}_{{\rm{MWIR}}}({\rm{I}}))+{{\rm{W}}}_{3}{(R}_{{\rm{LWIR}}}({\rm{I}})-{R}_{{\rm{LWIR}}}({\rm{M}}))$$

Here, I and M denote the I-state and M-state, respectively. ${R}_{{\rm{NIR}}-{\rm{SWIR}}}({\rm{I}})$ and ${R}_{{\rm{NIR}}-{\rm{SWIR}}}({\rm{M}})$ are the reflectances in the NIR-SWIR band before and after the phase transition; ${R}_{{\rm{MWIR}}}\left({\rm{I}}\right)$, ${R}_{{\rm{MWIR}}}\left({\rm{M}}\right)$ are the reflectances in the MWIR band; and ${R}_{{\rm{LWIR}}}\left({\rm{I}}\right)$, ${R}_{{\rm{LWIR}}}\left({\rm{M}}\right)$ are the reflectances in the LWIR band. The weighting factors W_1_, W_2_, and W_3_ for the NIR-SWIR, MWIR, and LWIR bands are set to 0.5, 1, and 1, respectively. Minimizing this FOM corresponds to high NIR–SWIR absorption in both states, together with state-selective high emissivity—high MWIR emissivity in the M-state and high LWIR emissivity in the I-state.

**Fig. 2:**
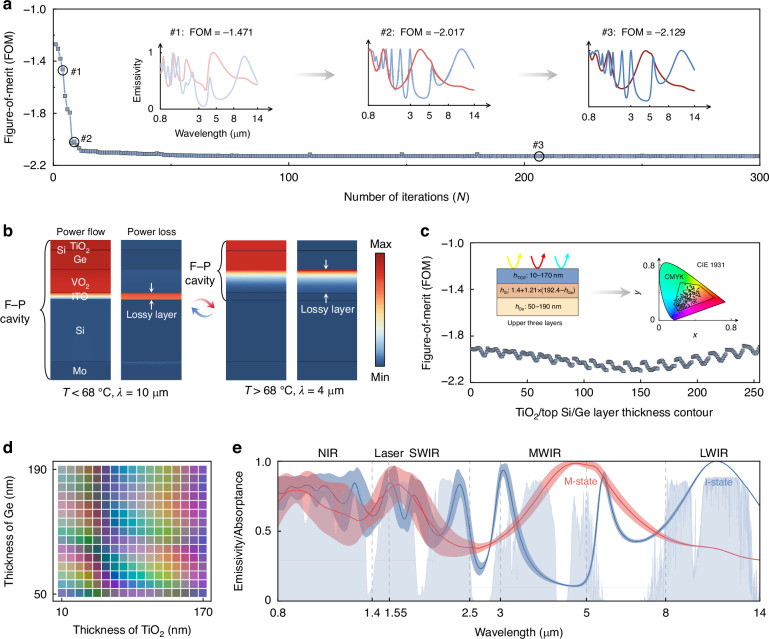
Optimization results and mechanism analysis of photonic structure. **a** Variation of the FOM as a function of the iteration number during the inverse-design process. The inset shows the emissivity spectra of the photonic structure at the initial (#1), intermediate (#2), and final (#3) stages under different states. **b** Distribution of power flow density and power loss for the inverse-designed photonic structure: at 10 μm before phase transition (left), and at 4 μm after phase transition (right). **c** The FOM as a function of the thicknesses of the top three layers of the photonic structure. The inset displays the coordinates of the VIS colors corresponding to the thickness variation of the top three layers. **d** Calculated VIS colors achieved by different thickness combinations of the TiO_2_ and Ge layers, with top-Si thickness constrained as described in the main text. **e** Calculated emissivity spectra of the structure before and after phase transition during the variation of the structural color layer thicknesses. The shaded regions represent the emissivity fluctuation caused by the change in VIS color

In addition, the thickness of the bottom metallic reflector, Mo, is fixed at 300 nm to ensure zero transmission (Fig. S1). Note that the VIS spectrum was initially excluded from the FOM. After the optimal infrared response was obtained, a separate mechanism was introduced to tune the VIS spectrum independently, as described later. Validation of the TMM accuracy, along with the optical constants of all material layers, is provided in Figs. S2 and S3^36–42^. As shown in Fig. 2a, the FOM rapidly decreased with increasing iteration number, reaching an apparent global minimum after approximately 67 iterations—this process required only 155 seconds on a standard personal computer (see Methods for detailed specifications). The inset in Fig. 2a illustrates the emissivity spectra at different stages of iteration. In the initial stage (#1), the dynamic tunability of the mid-infrared spectrum was limited. In the intermediate stage (#2), while the mid-infrared tunability improved, the absorption in the SWIR band was suboptimal. The final stage (#3) exhibits a balanced spectral performance: high absorption is maintained in the NIR and SWIR bands for both I-state and M-state, high selective emissivity is achieved in the LWIR for the I-state, and high selective emissivity is achieved in the MWIR for the M-state. The optimal variable set for the thicknesses of the layers (in nm) was determined to be (104.1, 1.4, 192.4, 235.7, 73.5, 620.2, 300.0). To elucidate the principle of dynamic mid-infrared spectral tuning, we plotted the power flow density and power loss distribution when a 10 μm wavelength (the center of the LWIR band) light is incident on the I-state structure, as depicted in Fig. 2b (left). An F-P resonance is excited, where the bottom Mo and Si layers act as the metallic mirror and the lossless dielectric spacer, respectively. The ITO layer functions as the lossy layer, absorbing the localized energy, thereby resulting in LWIR selective high emissivity. Following the phase transition to the M-state, light cannot penetrate the highly reflective metallic VO_2_ layer, effectively decoupling the incident light from the layers beneath VO_2_. The F-P resonance mode thus shifts to the top four layers (Fig. 2b, right). When 4 μm wavelength (the center of the MWIR band) light is incident on the M-state structure, a similar resonance occurs. In this configuration, the VO_2_ layer now acts as the lossy layer, absorbing localized energy, which yields MWIR selective high emissivity.

As the VIS reflectance was intentionally excluded from the initial FOM optimization, the resulting globally optimized structure, although delivering optimal spectral performance outside the VIS range, had a fixed VIS reflectance and therefore a single camouflage color. The materials for the top three layers (TiO_2_ /top Si/Ge) were chosen for several critical reasons: TiO_2_ and Si are highly effective for structural color generation; the VIS opacity of Ge ensures the structural color remains unaffected by the VO_2_ phase transition below it, guaranteeing optical robustness; and the similar infrared characteristics of top Si and Ge allow us to adjust their respective physical thicknesses while maintaining a relatively stable overall infrared optical thickness. This design rationale allows the thicknesses of the top three layers to be tuned to tailor the VIS spectrum while preserving the optimized infrared response. We varied the thicknesses of TiO_2_ and Ge within the ranges of 10–170 nm and 50–190 nm, respectively, subject to a constraint that the top Si thickness (*h*_top_Si_) was dependent on the Ge thickness (*h*_top_Si_ = 1.4 + 1.21×(192.4−*h*_Ge_)) to ensure the overall optical thickness of the top Si/Ge layer pair remained constant. By calculating 255 thickness combinations (with a 10 nm step) across these constraints, we generated a diverse palette of VIS colors (Fig. 2c inset and 2d). Quantitatively, this achievable color palette covers 66% of the standard CMYK color gamut, accommodating a wide range of VIS camouflage scenarios. The average FOM during this color modulation process was −2.00 ± 0.06, very close to the global optimum value of −2.13 (Fig. 2c). The corresponding infrared emissivity spectra for the 255 thickness combinations, presented in Fig. 2e (the shaded regions represent the emissivity fluctuation caused by the change in VIS color), reveal that altering the VIS color modifies the structure’s absorptivity from the VIS to the SWIR band—a feature beneficial for broadband optical camouflage. By contrast, this color tuning has almost no effect on the spectra within the MWIR and LWIR thermal-camouflage bands. This spectral decoupling capability is essential for achieving effective optical camouflage across diverse scenarios while maintaining stable thermal camouflage performance. Furthermore, the calculation results presented in Figs. S4 and S5 confirm that the proposed photonic structure is angle- and polarization-insensitive, a property critical for practical deployment.

### Fabrication and characterization

**Fig. 3:**
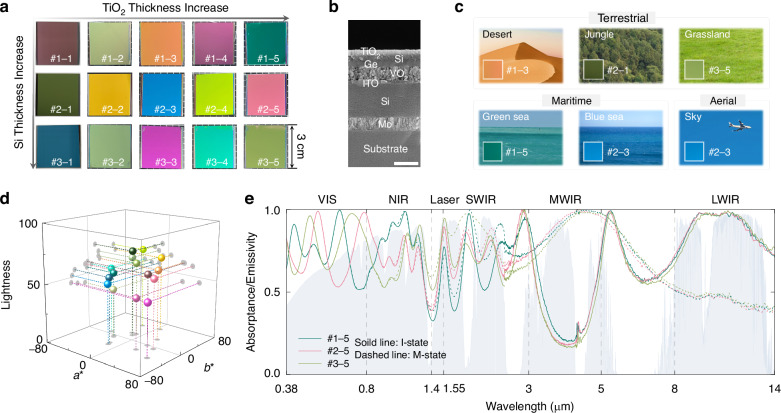
Fabricated samples and multispectral characterization. **a** Optical photographs of the fabricated photonic structures with varying thicknesses of the top TiO_2_ /top Si/Ge layers. **b** SEM cross-sectional image of sample #1-4 shown in (**a**). **c** VIS camouflage performance of the samples from Fig. 3a in diverse scenarios across terrestrial, maritime, and aerial environments. **d** Distribution of the CIE L*a*b* values derived from the measured VIS reflectance spectra of all samples shown in (**a**). **e** Measured absorption/emissivity spectra of three representative photonic structures (#1-5, #2-5, and #3-5) at I- and M-states

The proposed photonic structure was fabricated by magnetron sputtering. As shown in Fig. 3a, varying the thicknesses of the top TiO_2_/top Si/Ge stack yielded a rich variety of structural colors, closely matching our theoretical predictions. We designed and fabricated 15 samples (each 3 × 3 cm) corresponding to distinct colors (detailed structural parameters, listed as nominal thicknesses from the deposition rate and time, are provided in Table S1). The scanning electron microscopy (SEM) cross-section (Fig. 3b) and the energy-dispersive X-ray spectroscopy (EDS) elemental mapping (Fig. S6) of sample #1-4 clearly confirm the high-quality fabrication of the designed seven-layer photonic structure. These tailored structural colors cover a wide range of typical camouflage scenarios corresponding to terrestrial, maritime, and aerial environments (Fig. 3c). For instance, the orange-yellow sample (#1-3) is suitable for desert camouflage; the dark green (#2-1) and light green (#3-5) samples apply to jungle and grassland camouflage, respectively; the deep green (#1-5) sample is effective for green ocean environments; and the sky blue (#2-3) sample is applicable for both blue ocean and sky camouflage.

The CIE L*a*b* values (Fig. 3d) and the color coordinates in the CIE chromaticity diagram (Fig. S7) calculated from the measured reflectance spectra of these 15 samples reveal that the color points span a considerable region of color space. This demonstrates that the structural-coloration approach offers a high degree of freedom in customizing the brightness, hue, and saturation of the camouflage color. To assess the impact of color variation on the infrared spectrum, we present the absorption/emissivity spectra of three representative samples (#1-5, #2-5, and #3–5) under the I- and M-states, respectively (Fig. 3e, the spectra for the remaining samples are shown in Fig. S8).

The measured spectra confirmed several critical performance characteristics: First, the structural phase transition does not affect the VIS color (evidenced by the complete overlap of the VIS absorption curves before and after the transition), which is crucial for achieving stable VIS camouflage. Second, although color customization slightly modulates the NIR/SWIR absorption, the structure maintains an overall high absorption. The average NIR-SWIR absorption rates for the three colors (Fig. 3e) under the I-state were 0.67, 0.71, and 0.71, and under the M-state were 0.74, 0.73, and 0.78, respectively. This observed variation in NIR/SWIR absorption is, in fact, advantageous for nighttime optical stealth, a benefit demonstrated in the following section. Third, the customization of VIS color has a negligible impact on the MWIR and LWIR emissivity. The average MWIR/LWIR Planck-weighted band-averaged emissivities (computed at 25 °C for the I-state and 80 °C for the M-state) for the three colors under the I-state were 0.40/0.89, 0.40/0.89, and 0.34/0.89, respectively. After phase transition to the M-state, these average MWIR/LWIR emissivities shifted to 0.96/0.45, 0.96/0.45, and 0.95/0.46, respectively. Statistical analysis across all 15 fabricated samples reveals a consistent emissivity transition, with average MWIR/LWIR values shifting from 0.41 ± 0.04/0.90 ± 0.01 (I-state) to 0.93 ± 0.03/0.45 ± 0.01 (M-state). These results confirm the excellent opto-thermal spectral decoupling capability of the proposed structure.

To verify the adaptability of the design, we fabricated a flexible sample on a polyimide (PI) substrate with an identical layer configuration to sample #1-1 (Fig. S9). This flexible sample demonstrates excellent conformal adhesion to curved surfaces, and the measured reflection spectra indicate that adopting a flexible form factor does not compromise optical performance. This demonstration is critical for assessing the feasibility of practical deployment, as real-world camouflage targets often involve complex or curved geometries.

### Demonstration of opto-thermal camouflage

**Fig. 4:**
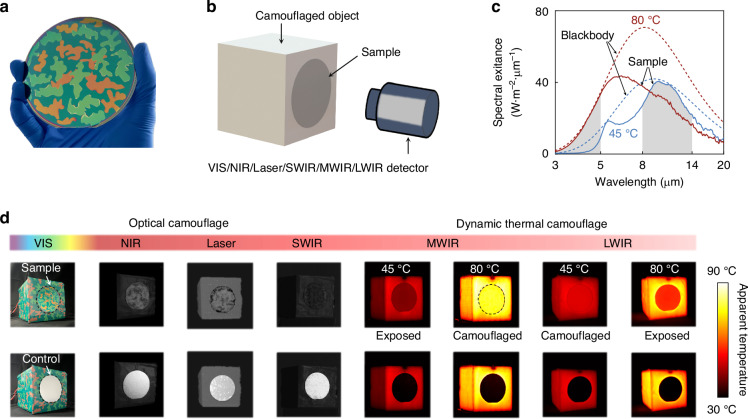
Experimental demonstration of broadband opto-thermal camouflage performance. **a** Optical photograph of the 4-inch photonic structure fabricated via the mask-assisted process. **b** Schematic diagram of the experimental setup used to characterize the opto-dynamic thermal camouflage performance of the sample. **c** Calculated spectral radiant exitance of the sample before and after phase transition (corresponding to 45 °C and 80 °C, respectively), compared to a blackbody at the same temperatures. **d** Images of the cubic model affixed with the sample shown in (**a**), captured using VIS, NIR, laser, SWIR, MWIR, and LWIR detectors. The MWIR and LWIR images were specifically captured at two distinct temperatures (45 °C and 80 °C)

To demonstrate the opto-dynamic thermal camouflage performance, we fabricated patterned camouflage samples on silicon wafers by shadow masking, with patterns selected for visual similarity to natural backgrounds. Note that the colors and patterns were designed for a proof-of-concept demonstration to validate the material’s capabilities, rather than strictly adhering to current military camouflage specifications. Detailed structural parameters for the different colors within the pattern, along with other patterned samples, are provided in Tables S2–S4 and Fig. S10. As shown in Fig. 4a, the sample was then integrated onto a cubic model simulating a camouflaged object to validate its multispectral performance (Fig. 4b). The surface of the object was covered with a sticker featuring the same camouflage pattern as the sample. This setup, capable of precise temperature regulation, was designed to establish a standardized environment with controllable thermodynamic parameters, thereby verifying the temperature-selective thermal camouflage capability of the sample.

We first calculated the spectral radiant exitance of the sample before and after the phase transition (Fig. 4c). In the I-state, the sample’s LWIR spectral radiant exitance matches that of a blackbody at the same temperature. After transitioning to the M-state, this blackbody-matching characteristic shifts to the MWIR band. This shift is the key to dynamically controlling the photonic structure’s thermal exposure under the two distinct thermal detectors. Figure 4d demonstrates the multispectral camouflage performance, with low-emissivity aluminum foil serving as a reference. As a practical benchmark, we also evaluated the performance against traditional military camouflage fabric, with comparative results presented in Fig. S11.

Under a VIS detector, the camouflage pattern blends seamlessly with the object’s surface. As the detection wavelength increases to NIR, laser, and SWIR, the pattern on the object’s surface disappears, whereas the sample retains its camouflage pattern. This resilience, originating from the broadband reflectance modulation of the structural color, is crucial for nighttime optical camouflage against complex backgrounds. As the detection band extends into the thermal spectrum, we demonstrate dynamic thermal camouflage capabilities in the I-state (sample at 45 °C) and M-state (sample at 80 °C). Under an MWIR detector, the I-state sample is easily detected, whereas the M-state sample blends with the object’s surface. Conversely, under an LWIR detector, the I-state sample blends with the object’s surface, but becomes exposed after transitioning to the M-state. In stark contrast, the control sample is easily detected under all optical and thermal detectors.

The temperature- and spectral-selective nature of this thermal camouflage renders it more suitable for specific heated targets than for ambient environments. Under ambient conditions, the active heating mode should be avoided whenever possible, or the thermal communication function should be enabled via a short-burst mode only when necessary. This is because an increase in physical temperature inevitably leads to higher radiance, turning the device into a “hotspot” relative to the background and increasing the risk of exposure. Quantitative evaluations of the opto-thermal camouflage performance presented in Fig. 4d are detailed in Figs. S12 and S13. To verify the calculated wide-angle stability of the structure, we demonstrated the opto-thermal camouflage effectiveness of the sample under various viewing angles (Fig. S14).

While these images captured by consumer or commercial-grade detectors validate the physical mechanism of our proposed camouflage strategy, there remains a performance gap compared with high-sensitivity or high-resolution military detectors. Consequently, practical performance against more advanced sensors requires further verification. Tables S5–S7 provide detailed performance specifications for all detectors used, clarifying the conditions under which our current assessment is valid. Moreover, visible camouflage based on human perceptual color similarity may face detection risks from multispectral or hyperspectral sensors, which rely on the spectral fingerprints of object surfaces rather than mere apparent color contrast. Advanced optical camouflage may require more refined structural designs to achieve rigorous spectral fingerprint matching.

Additionally, although this work focuses on opto-thermal camouflage, microwave radar remains a cornerstone of military reconnaissance. A promising future direction involves introducing a hierarchical structural design strategy^29^. By extending the current configuration into a “top-layer transparent/bottom-layer absorbing” architecture coupled with an underlying radar absorber, the system could be endowed with multispectral camouflage capabilities.

### Demonstration of encrypted information transfer

**Fig. 5:**
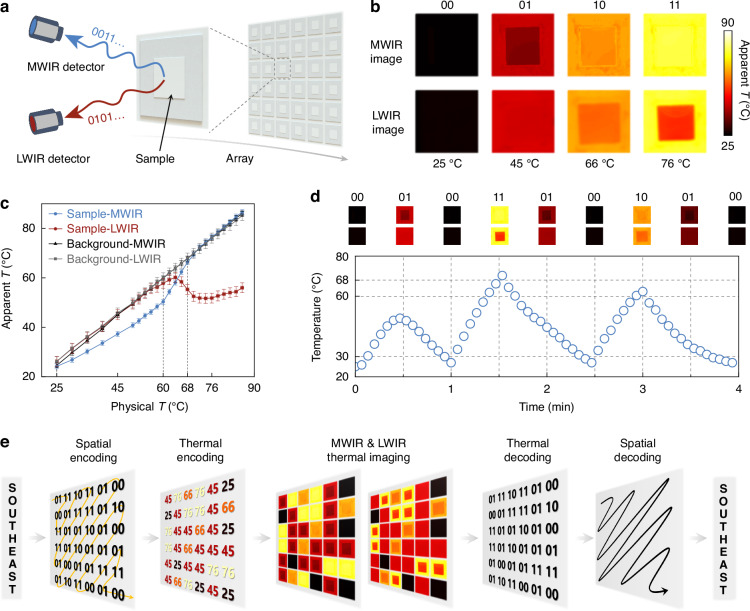
Experimental demonstration of encrypted information transfer. **a** Schematic of encrypted information transmission based on controlling the photonic structure’s MWIR/LWIR thermal exposure, illustrating the progression from a single unit to an arrayed configuration. **b** MWIR/LWIR images of a single unit at different temperatures, with four encoding states defined according to the sample’s four distinct thermal-exposure levels. **c** Recorded MWIR/LWIR apparent temperatures of the sample and background regions for a single unit as a function of physical temperature. **d** Time-domain response characteristics of a single unit cell under continuous temperature modulation, with a switching period of 30 s. **e** Conceptual diagram for the encrypted transmission of the word “SOUTHEAST” using a 6 × 6 array

Dynamically controlling an object’s thermal exposure across two distinct thermal bands simultaneously offers new possibilities for encrypted information transmission. Figure 5a illustrates this concept, in which individual units are temperature-controlled to transmit information across the MWIR and LWIR bands. The principle of this encoding is shown in Fig. 5b, which displays the MWIR/LWIR images of a single unit cell at different temperatures. A high-emissivity coating serves as the background to provide the thermal contrast required for decoding (Fig. S15). This configuration allows the temperature-induced modulation of the sample’s MWIR and LWIR emissivity to generate four distinct thermal exposure states relative to the high-emissivity background.

These four states result directly from the emissivity modulation and are quantified by the apparent-temperature plots in Fig. 5c. At ambient temperature (pre-heating), the radiance difference is minimal, rendering the sample imperceptible and thus concealed in both bands. As the temperature increases (30–56 °C), the background’s apparent MWIR/LWIR temperatures rise accordingly. The sample’s selective high LWIR emissivity causes its apparent LWIR temperature to track the background (remaining concealed), whereas its low MWIR emissivity results in a significantly lower apparent MWIR temperature (creating contrast). This divergence exposes the sample only in the MWIR band. When the temperature enters the phase transition region (56–68 °C), the apparent MWIR temperature difference begins to decrease, while the apparent LWIR temperature progressively deviates from the background, exposing the sample in both bands. Finally, above 68 °C (post-transition), the sample’s selective high MWIR emissivity matches its apparent temperature to the background (concealed in MWIR), while its low LWIR emissivity creates significant thermal contrast, exposing the sample only in the LWIR band.

The efficacy of this decoding method, which relies on resolving individual thermal pixels, is governed by the relevant observation parameters. This parameter space is defined by factors such as unit cell dimensions, array pitch, and detector performance. For instance, using the MWIR and LWIR detectors employed in this study, the reliable decoding distance for a single 3 × 3 cm thermal pixel ranges from 0.3 to 16.6 m in the MWIR and from 0.36 to 2.7 m in the LWIR (Fig. S16). This difference is detector-limited rather than intrinsic to the structure. Because thermal communication involves frequent temperature switching, cyclic stability is critical. We subjected sample #1-5 (Fig. 3a) to 1,200 heating-cooling cycles between 20 and 85 °C, measuring its spectra every 200 cycles. The results indicate negligible spectral degradation, confirming the reliability of the device (Fig. S17).

To encode thermal exposure levels into information, the four distinct states shown in Fig. 5b are defined as binary codes: ‘00’ (concealed), ‘01’ (exposed only in MWIR), ‘10’ (exposed in both), and ‘11’ (exposed only in LWIR). To validate the dynamic information transmission capability, we characterized the time-domain response of a single unit in Fig. 5d. Driven by the response limits of the temperature controller, the switching interval was set to 30 s (Fig. S18). This specific interval also functions as a communication security key, as accurate information retrieval requires strict temporal synchronization between the transmitter and receiver. Constrained by the communication rate, the current prototype is best suited for low-bandwidth, high-security short-message interactions, such as identification codes or static password transmission.

Scaling up to an array configuration offers a viable pathway to further enhance the communication rate. Figure 5e illustrates this concept, in which the thermal array image is synthesized from the different encoding states of a single unit. Building such a system is technically feasible: for example, independent temperature control of individual units can be achieved using a matrix-addressing circuit combined with a multi-channel PID controller, while thermal crosstalk can be suppressed using insulation materials, as confirmed by the theoretical model in Fig. S19.

In the demonstrated arrayed transmission, each letter of the message “SOUTHEAST” is converted into an 8-bit binary code, which is then encoded by four unit cells. A key advantage of this arrayed configuration is enhanced encryption via spatial encoding. We spatially encode the message onto a 6 × 6 array following a pre-determined diagonal serpentine path, which serves as an additional encryption key. Subsequently, the binary code for each unit along this path is thermally encoded via temperature control. Decrypting this multi-level encrypted information requires multiple keys. First, the complete thermal signature must be acquired using both MWIR and LWIR detectors. This signature is then decoded into a binary array based on the pre-defined encoding rules. Finally, the correct information is retrieved only by assembling the binary data following the specific spatial path. This multi-level encryption method—simultaneously incorporating the wavelength, temperature, and spatial domains—exponentially increases the difficulty of decryption, offering a new avenue for advanced encrypted information transmission.

To assess practical viability, we established a theoretical model to estimate the energy budget of the thermal communication system (Fig. S20). Assuming an array periodicity of 5×5 cm and a thermal pixel size of 3 × 3 cm, calculations indicate that under natural convection, the power required to heat a single unit from room temperature to 68 °C is approximately 2.5 W. Consequently, scaling to large-area arrays or maintaining continuous operation would necessitate a continuous power supply. Potential strategies to reduce energy consumption include miniaturizing the unit cell periodicity and lowering the intrinsic phase transition temperature of VO_2_ via elemental doping. The former directly reduces the effective heating area, while the latter minimizes the temperature swing required to switch between encoding states—a strategy that would simultaneously enhance the thermal communication rate and mitigate thermal crosstalk between units.

## Discussion

In summary, we have presented a photonic structure, designed through a Bayesian-optimization-based inverse approach, that brings broadband opto-thermal camouflage and infrared encrypted communication together within a single platform. The key is the spectral decoupling built into the architecture: structural color in the visible band can be tuned independently of the NIR–SWIR absorption, and both remain undisturbed when the VO₂ layer modulates MWIR and LWIR emissivity. This resolves a tension that has constrained earlier multispectral materials, where adjusting one band almost inevitably perturbed another. The platform also has clear limitations. Robustness against high-resolution military-grade sensors and hyperspectral imaging still needs to be established, and extending the concealment to the radar band will be an important direction for future work. The present switching rate, set by the thermal response of the unit, restricts the current prototype to low-bandwidth, high-security messaging; reducing the unit-cell size and lowering the VO₂ transition temperature through elemental doping should shorten the thermal cycle and ease the heating power required for scaling. Beyond the camouflage scenarios shown here, the same design principle suggests possibilities in low-observable thermal management, cross-band anti-counterfeiting, and short-range covert links where radio-frequency channels are denied—indicating that stealth and communication need not be designed as opposing functions when their spectral channels are properly separated.

## Materials and methods

### Numerical simulations and inverse design

The distribution of power flow and power loss shown in Fig. 2 was visualized using the finite element method within COMSOL Multiphysics software, employing two-dimensional periodic boundary conditions. The complex refractive indices used in the calculations for TiO_2_, Si, Ge, VO_2_, ITO, and Mo were taken from the literature^36–42^ (Fig. S2). To ensure the accuracy of the simulations, we also used COMSOL to calculate the emissivity spectrum corresponding to the final optimization stage in Fig. 2a and compared it with the results obtained from the TMM used during the BO-based inverse-design process. The excellent agreement between the two calculation methods validates the accuracy of the power flow and power loss distributions computed via COMSOL. The BO-based inverse-design process was executed in the MATLAB environment on a system running Windows 11 Pro, version 24H2. The computations were performed on hardware equipped with an Intel Core i5-13400F processor, 64 GB of RAM, and an NVIDIA GeForce GTX 1650 Super GPU.

### Fabrication of the photonic structure

All rigid samples were fabricated on 0.5-mm-thick Si wafers (Beijing Shengyakang Technology Co., Ltd.); the flexible sample was fabricated on a PI substrate. Prior to deposition, the substrates were sequentially cleaned by ultrasonication in ethanol, acetone, and deionized water for 15 min each, and then dried under a stream of high-purity nitrogen. All layers were grown by magnetron sputtering. The VO_2_ layer was deposited from a 4-inch V_2_O_3_ target, while the remaining layers were deposited from 2-inch targets of the corresponding materials. The deposition parameters for each layer are listed in Table S8. After VO_2_ deposition, the sample was annealed in situ under 5 Torr for 5 min and then cooled naturally before the subsequent layers were deposited; the annealing temperature was 450 °C for samples on Si wafers and 375 °C for the flexible sample on PI.

### Optical properties characterization

Cross-sectional images of the sample were obtained using a field emission SEM (Regulus 8100, Hitachi). The elemental distribution was analyzed using EDS (Ultim Max 65, Oxford). The reflectance (*R*) and transmittance (*T*) spectra of the samples in the 3–14 µm range were obtained using a Fourier transform infrared spectrometer (Nicolet iS50, Thermo Fisher Scientific) equipped with a gold-plated integrating sphere with an angle of incidence of 12°. The absorptivity was calculated as *A* = 1 − *R* − *T*; by Kirchhoff’s law of thermal radiation, the absorptance equals the emissivity. The VIS, NIR, and SWIR reflectance spectra were measured by an ultraviolet-visible-near-IR spectrophotometer (UV-3600, Shimadzu) equipped with an integrating sphere at an incident angle of 8°.

The band-averaged emissivities reported in this work are computed as Planck-weighted averages:

1 $${\bar{\varepsilon }}_{{\rm{band}}}(T)=\frac{{\int }_{{\lambda }_{1}}^{{\lambda }_{2}}\varepsilon (\lambda ){I}_{{\rm{BB}}}(\lambda ,T)d\lambda }{{\int }_{{\lambda }_{1}}^{{\lambda }_{2}}{I}_{{\rm{BB}}}(\lambda ,T)d\lambda }$$

where ${I}_{{\rm{BB}}}(\lambda ,T)$ is the Planck blackbody spectral radiance and [${\lambda }_{1},{\lambda }_{2}$] denotes the corresponding spectral band (3–5 μm for MWIR and 8–14 μm for LWIR). The weighting temperature is taken as 25 °C for the I-state and 80 °C for the M-state, representing the material’s spectrally-averaged emissivity (Planck-weighted) before and after the VO₂ phase transition.

### Multispectral image capture

Multispectral images of the samples were captured across the VIS, NIR, laser, SWIR, MWIR, and LWIR bands using a combination of detectors: an iPhone 13 Pro (Apple) for the VIS band; an OV1080P_PCBA_NIR detector (Zhongweiaoke Inc.) for the NIR band; an OWL-640S camera (ISUZU OPTICS) for the SWIR band; a FARO Focus S350 system (FARO Technologies, Inc.) for the 1.55 μm laser wavelength; a FLIR 6700 detector (FLIR) for the MWIR band; and a FLIR E5 Pro detector (FLIR) for the LWIR band.

### VIS color display

The measured VIS reflectance spectra were converted into colors using a CIE-based model. The tristimulus values *X*, *Y*, and *Z* in the CIE color space were calculated by integrating the spectral reflectance with the CIE color-matching functions, which represent the spectral sensitivity of human color vision, according to the following equations^43–45^:

2 $$X=100\frac{\int I\left(\lambda \right)r\left(\lambda \right)\bar{x}\left(\lambda \right)d\lambda }{\int I\left(\lambda \right)\bar{y}\left(\lambda \right)d\lambda }$$

3 $$Y=100\frac{\int I\left(\lambda \right)r\left(\lambda \right)\bar{y}\left(\lambda \right)d\lambda }{\int I\left(\lambda \right)\bar{y}\left(\lambda \right)d\lambda }$$

4 $$Z=100\frac{\int I\left(\lambda \right)r\left(\lambda \right)\bar{z}\left(\lambda \right)d\lambda }{\int I\left(\lambda \right)\bar{y}\left(\lambda \right)d\lambda }$$

where $I(\lambda )$ is the spectral power distribution of the light source and $r(\lambda )$ is the object’s reflectance. We used standard D65 illumination to represent outdoor lighting conditions. The tristimulus values *X*, *Y*, and *Z* are used to determine the brightness and chromaticity of a color (Fig. S21). The brightness is represented by the parameter *Y*, while the chromaticity is determined by the normalization parameter:

5 $$x=\frac{X}{X+Y+Z}$$

6 $$y=\frac{Y}{X+Y+Z}$$

7 $$z=\frac{Z}{X+Y+Z}$$

The color is determined by the chromaticity coordinate (x, y) in the chromaticity diagram, which, in turn, is determined by its reflection spectrum in the 1931 CIE-XYZ color system. To better describe the chromatic response of the human eye, the CIE-${L}^{* }{a}^{* }{b}^{* }$ color space is derived by a nonlinear transformation of the CIE-XYZ color space. Here, ${L}^{* }$ represents the lightness, ${a}^{* }$ represents the redness and greenness, and ${b}^{* }$ represents the yellowness and blueness. These parameters can be transformed from the tristimulus values *X*, *Y*, and *Z* numerically as:

8 $${L}^{* }=116f\left(Y/{Y}_{0}\right)-16$$

9 $${a}^{* }=500\left[f\left(X/{X}_{0}\right)-f\left(Y/{Y}_{0}\right)\right]$$

10 $${b}^{* }=200\left[f\left(Y/{Y}_{0}\right)-f\left(Z/{Z}_{0}\right)\right]$$

where ${X}_{0}$, ${Y}_{0}$, and ${Z}_{0}$ are tristimulus values corresponding to the color white and

11 $$f\left(t\right)=\left\{\begin{array}{l}{t}^{\frac{1}{3}},\,t > {\left(\frac{24}{116}\right)}^{3}\\ \left(\frac{841}{108}\right)t+\frac{16}{116},t\le {\left(\frac{24}{116}\right)}^{3}\end{array}\right.$$

## Supplementary information

Supplementary materials

## Data Availability

The data that support the findings of this study are available within this article and its supplementary materials.
