# Supplementary material for: Broadband opto-thermal camouflage and infrared encrypted communication via inverse design
Source: Light Sci Appl. 2026 Jun 22;15:275. doi: 10.1038/s41377-026-02370-x (PMC13287681; doi:10.1038/s41377-026-02370-x)
Supplement: Supplementary file 1 — Supplementary materials [file 41377_2026_2370_MOESM1_ESM.docx]

Supplementary Materials for

**Broadband opto-thermal camouflage and infrared encrypted communication via inverse design**

Qixiang Chen^1,^ ^†^, Chengcong Li^2, 3, †^, Zhuning Wang^4, †^, Zezhao Ju^5^, Jieren Song^5^, Hongtao Lin^5^, Huajie Tang^1^, Chengyue Guo^1^, Yaoguang Ma^4, *^, Xun Cao^2, 3, *^, Dongliang Zhao^1, 6, *^

^1^ School of Energy and Environment, Southeast University, Nanjing, Jiangsu 210096, China

^2^ State Key Laboratory of Functional Crystals and Devices, Shanghai Institute of Ceramics, Chinese Academy of Sciences, Shanghai 201899, China

^3^ Center of Materials Science and Optoelectronics Engineering, University of Chinese Academy of Sciences, Beijing 100049, China

^4^ State Key Laboratory for Extreme Photonics and Instrumentation, College of Optical Science and Engineering, Zhejiang University, Hangzhou, Zhejiang, 310013, China

^5^ The State Key Lab of Brain-Machine Intelligence, Key Laboratory of Micro-Nano Electronics and Smart System of Zhejiang Province, College of Information Science and Electronic Engineering, Zhejiang University, Hangzhou, Zhejiang, 310027, China

^6^ Institute of Science and Technology for Carbon Neutrality, Southeast University, Nanjing, Jiangsu 210096, China

^*^Corresponding author: mayaoguang@zju.edu.cn; cxun@mail.sic.ac.cn; dongliang_zhao@seu.edu.cn


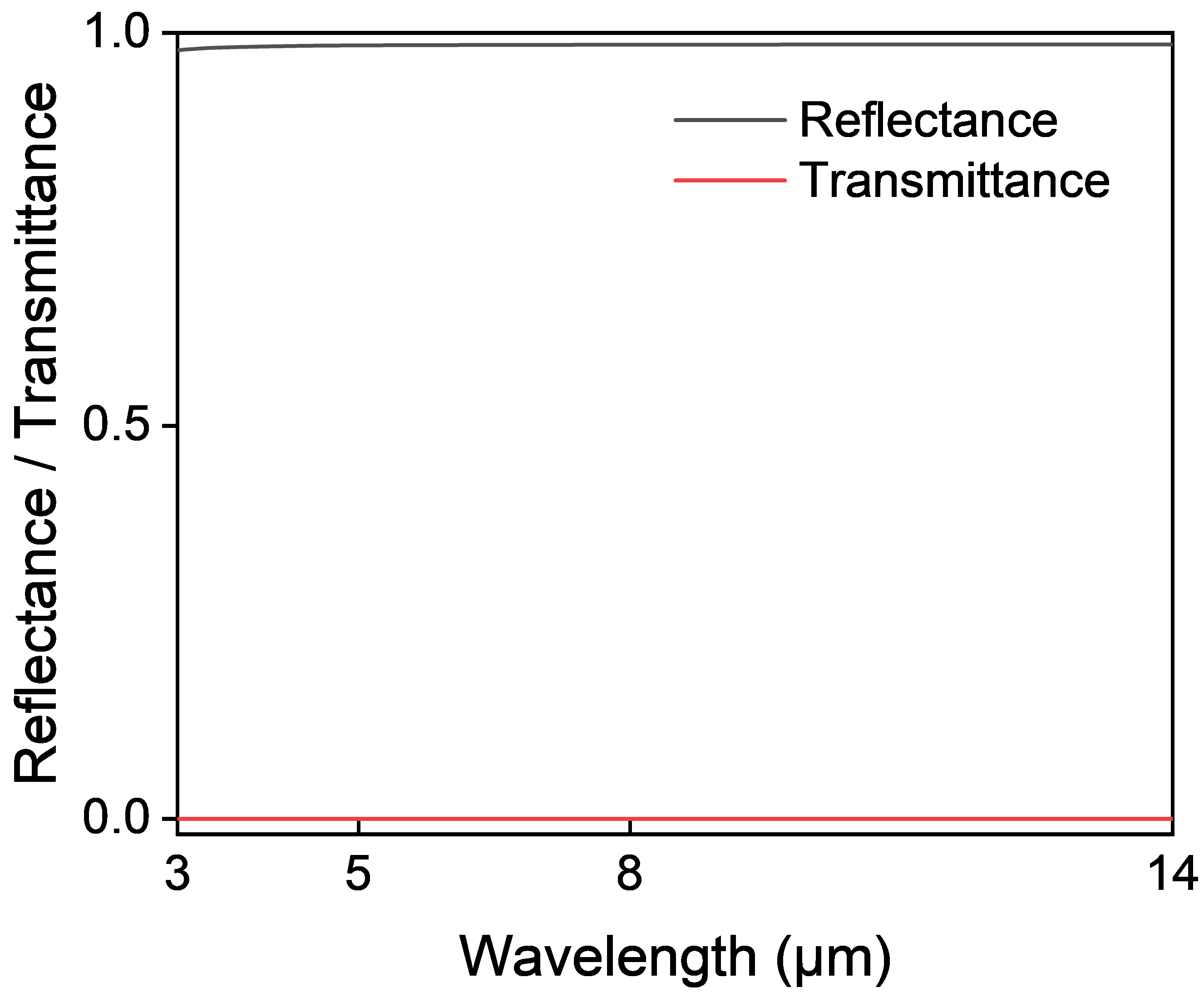


**Fig. S1** Calculated reflectance and transmittance spectrum of the 300-nm-thick Mo layer.


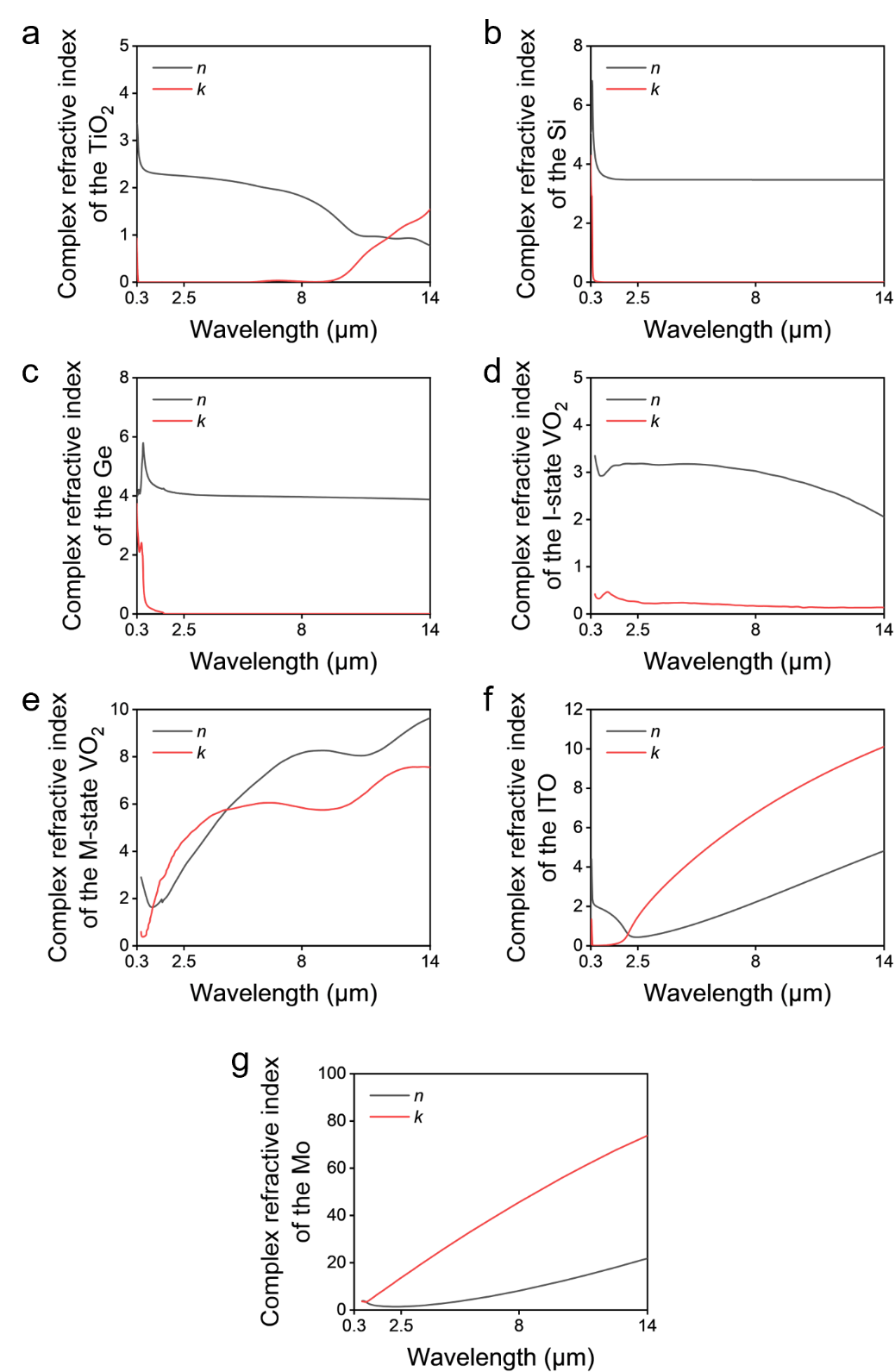


**Fig. S2** Refractive index and extinction coefficient of the TiO_2_ (a), Si (b), Ge (c), I- (d) and M-states VO_2_ (e), ITO (f), and Mo (g).


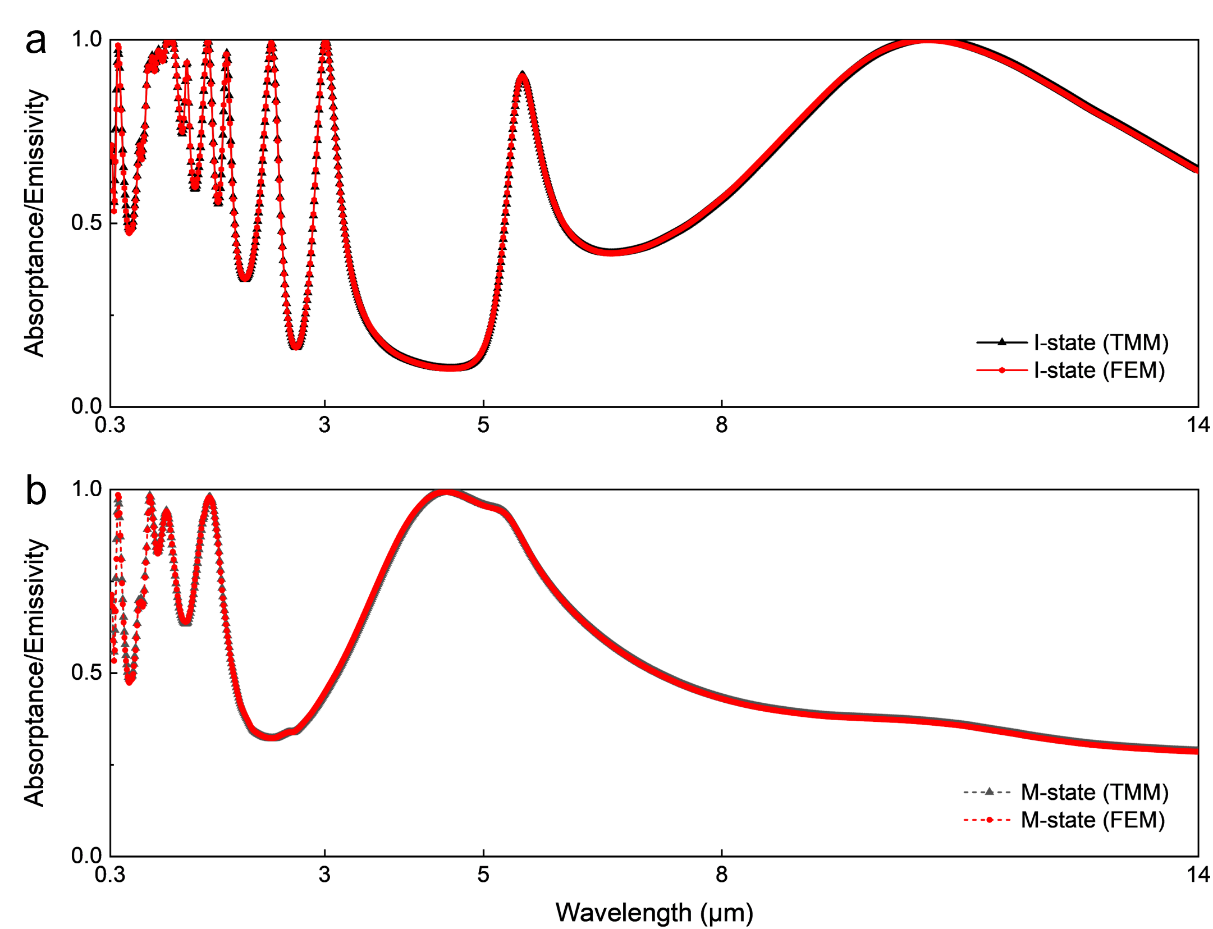


**Fig. S3** Calculated emissivity spectra of the photonic structure under the I- (a) and M-states (b) using the Transfer Matrix Method and the Finite Element Method, respectively. The layer thicknesses from top to bottom are 104.1, 1.4, 192.4, 235.7, 73.5, 620.2, and 300.0 nm.


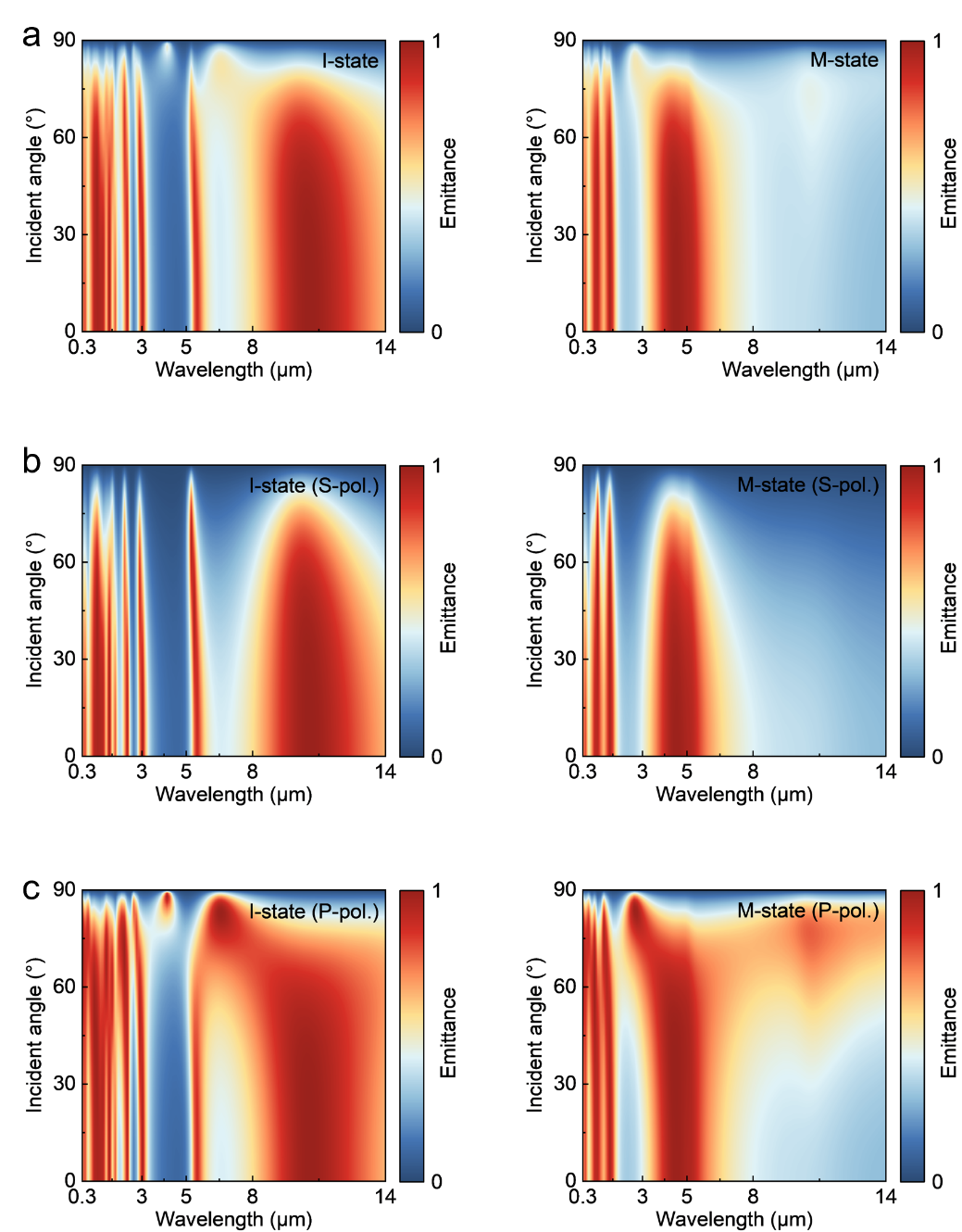


**Fig. S4** Calculated angle-dependent emissivity of the photonic structure in the I-state (left) and M-state (right) under: (a) non-polarized incidence; (b) S-polarization; and (c) P-polarization.


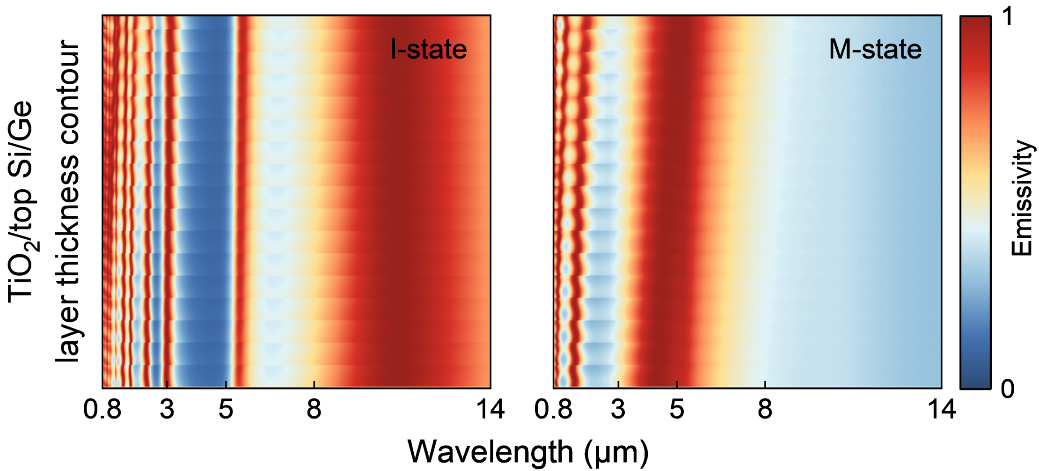


**Fig. S5** Calculated emissivity spectra corresponding to the 255 thickness combinations of the top three layers shown in Fig. 2c.


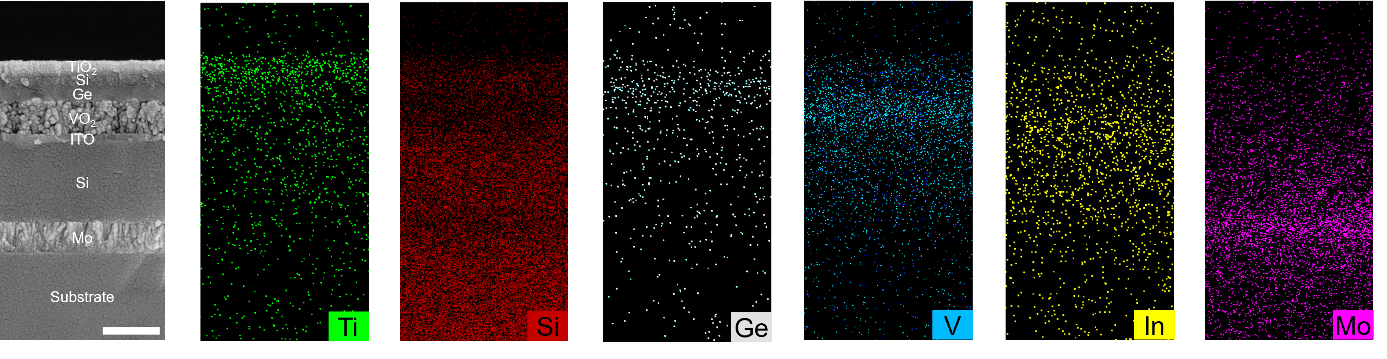


**Fig. S6** Elemental distribution map of the SEM cross-section region shown in Fig. 3b.


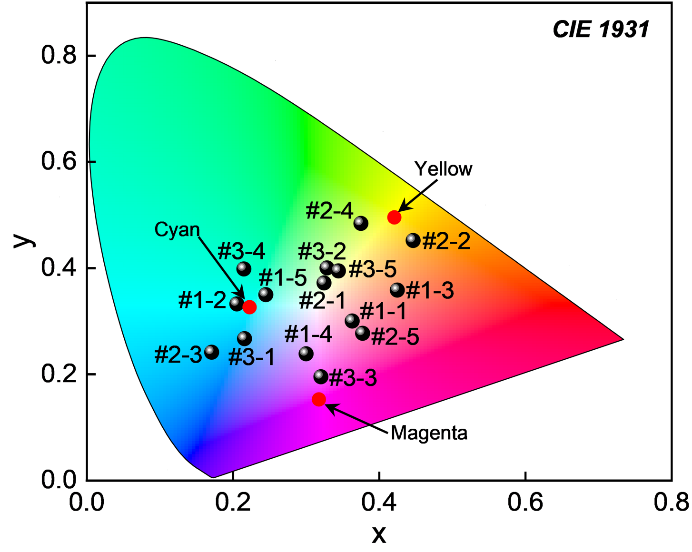


**Fig. S7** Distribution of the visible colors of the 15 samples displayed in Fig. 3a on the CIE 1931 chromaticity diagram.


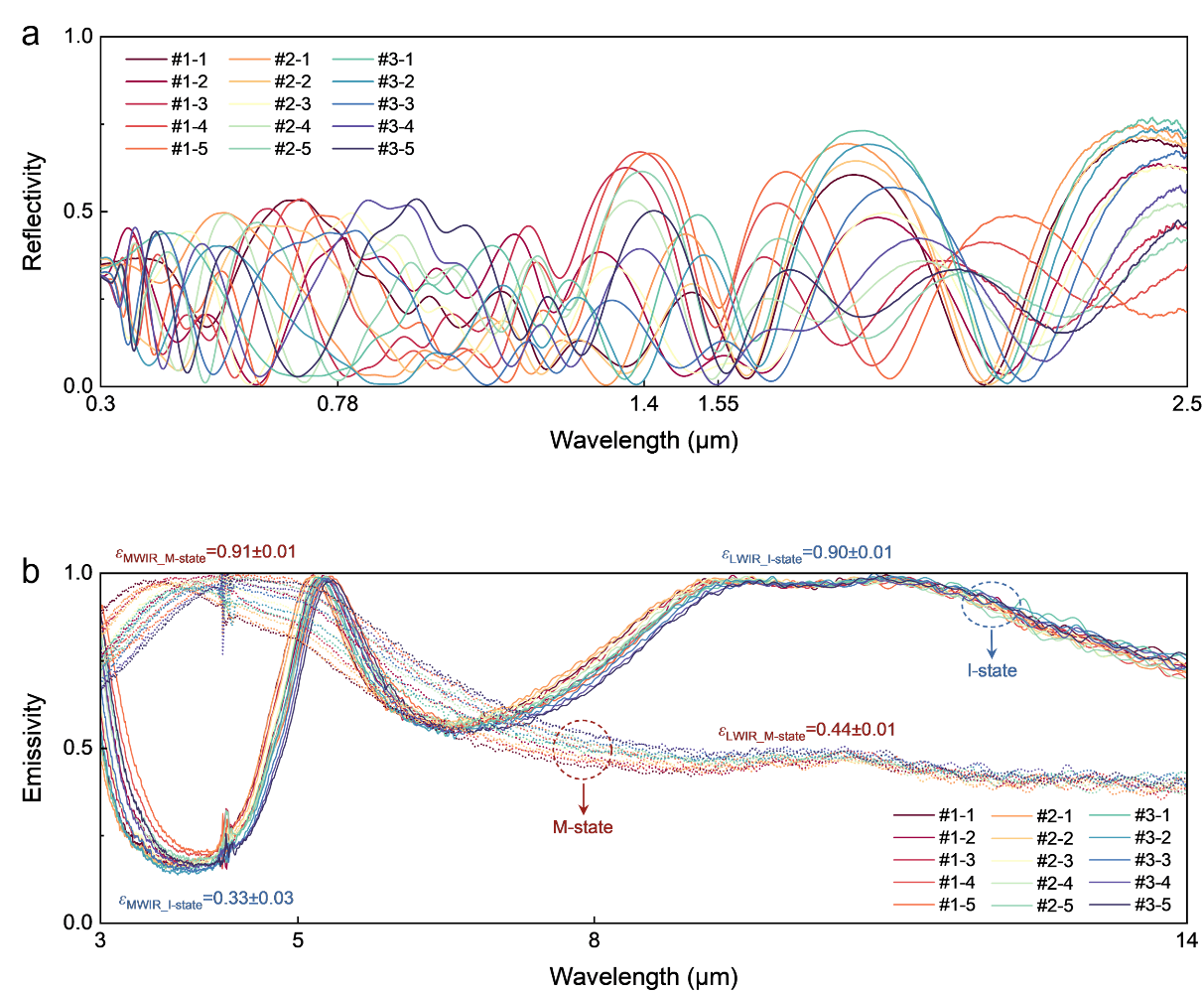


**Fig. S8** (a) Measured reflectance spectra (I-state) of the 15 samples shown in Fig. 3a; (b) Measured emissivity spectra of the 15 samples shown in Fig. 3a in the I-state (solid lines) and M-state (dashed lines).


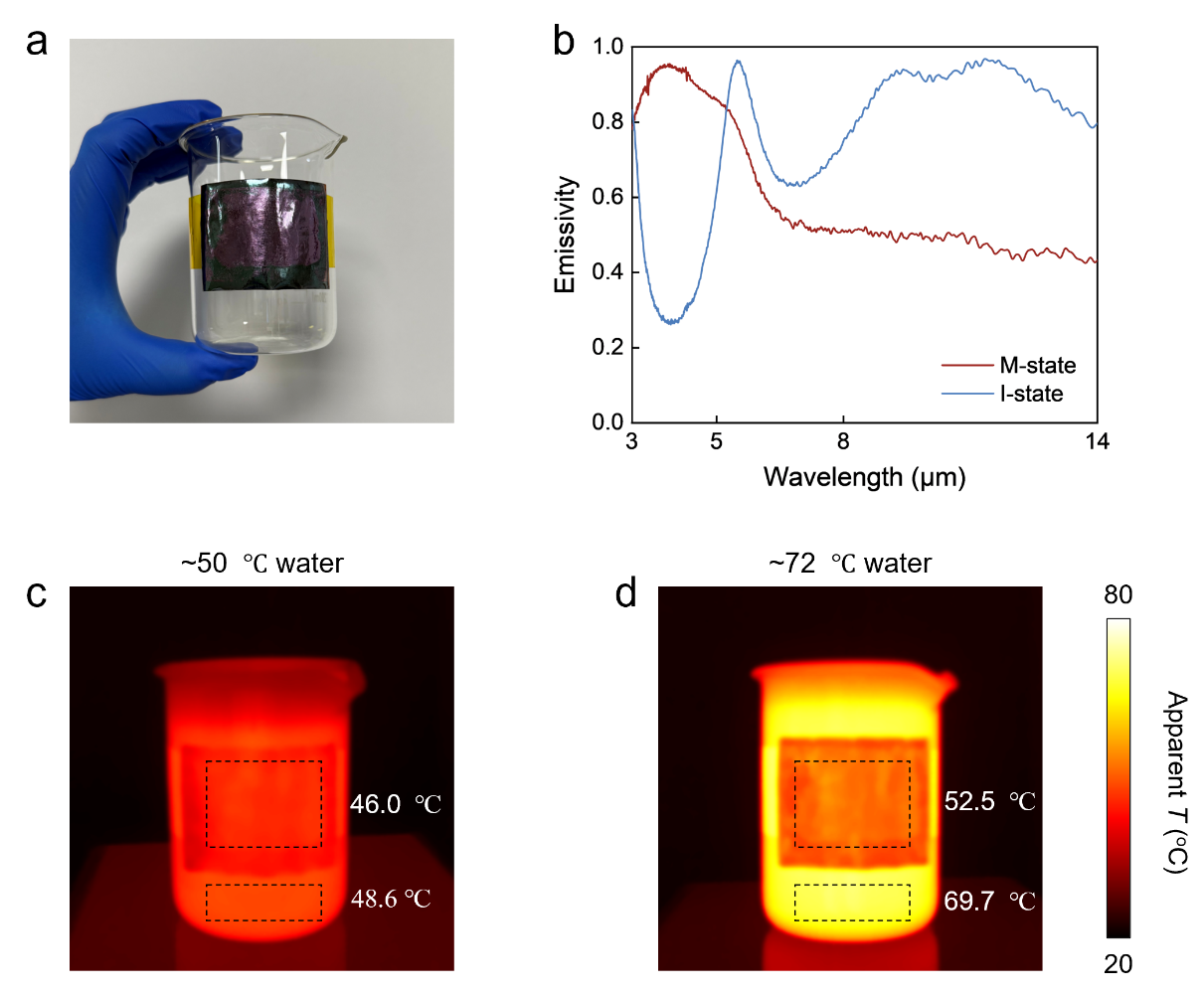


**Fig. S9** (a) Optical photograph of the flexible sample fabricated on a Polyimide (PI) substrate. (b) Measured emissivity spectra of the flexible sample before and after phase transition. (c) LWIR images of the flexible sample attached to the curved wall of a beaker filled with water at 50 (left) and 72 ℃ (right).


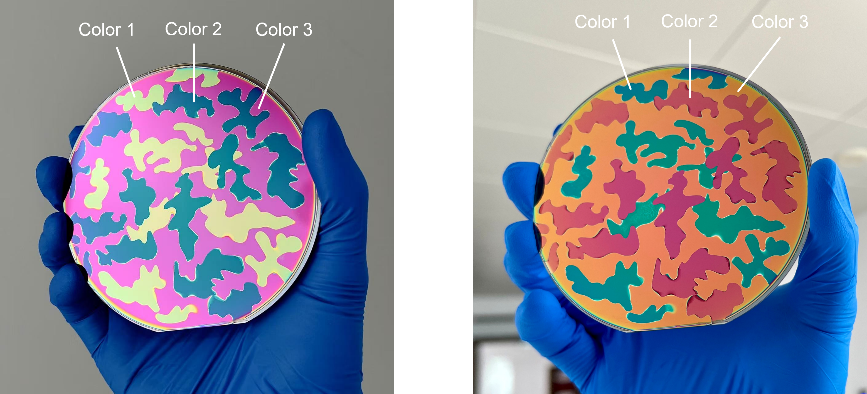


**Fig. S10** Fabricated photonic structure samples featuring different camouflage patterns from the one shown in Fig. 4a. The detailed thicknesses of the top three layers for the two samples are provided in Table S3 and Table S4, respectively.


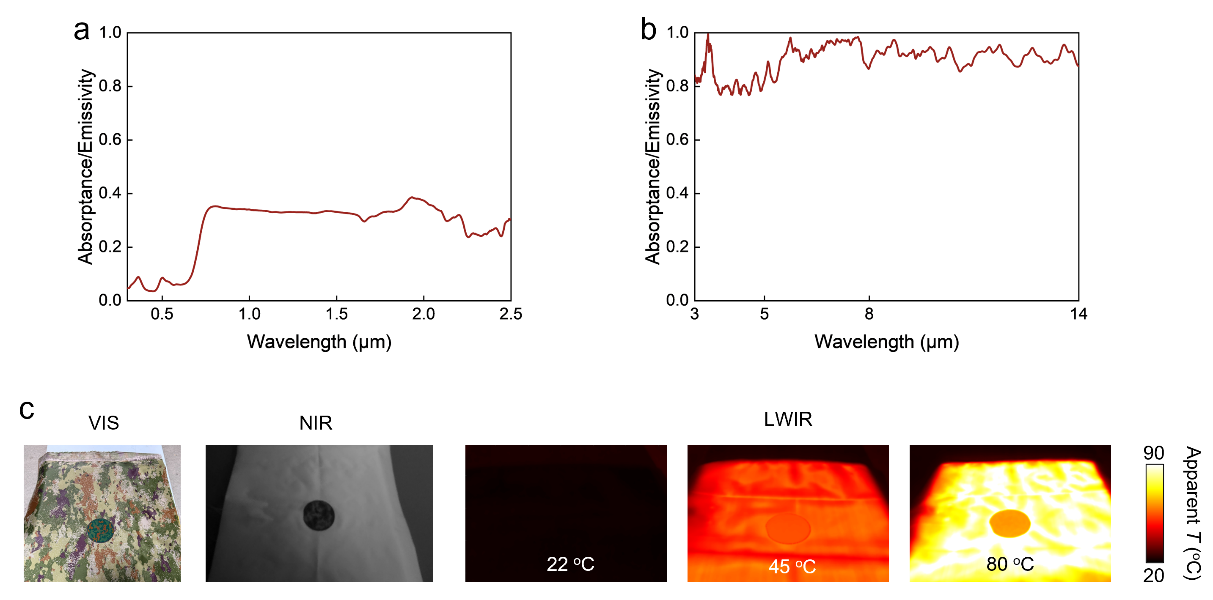


**Fig. S11** (a) and (b) Measured VIS-NIR-SWIR (a) and MIR (b) bands absorptance/emissivity spectra of the traditional camouflage fabric. (c) Comparative optical, NIR, and LWIR (pre- and post-phase transition) images of the patterned sample and a traditional commercial military camouflage fabric.


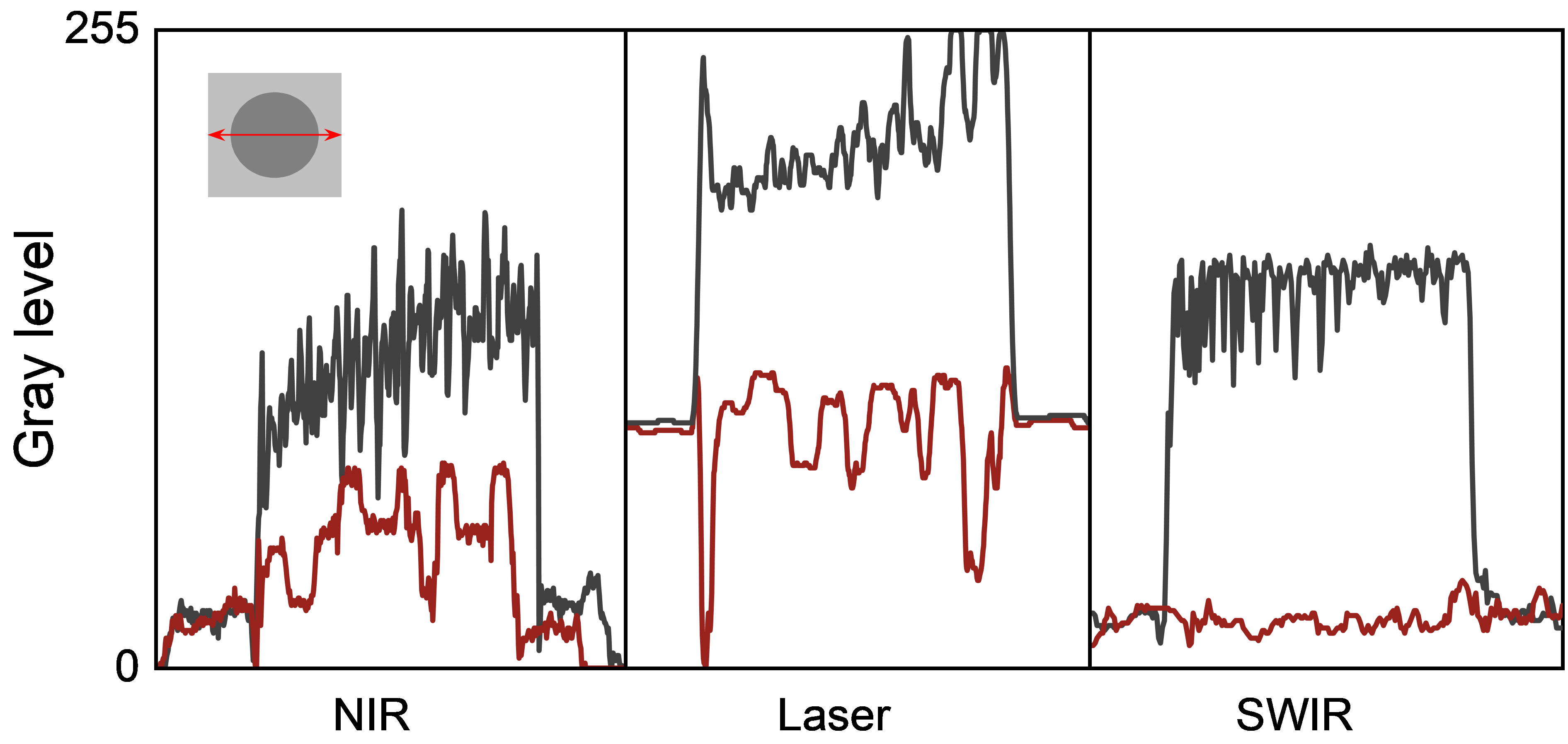


**Fig. S12** Line profiles of the grayscale intensity across the cross-sectional area analyzed from the images captured by the NIR, laser, and SWIR detectors in Fig. 4d. (The inset illustrates the location of the line profiles; higher grayscale values indicate stronger light reflection signals).


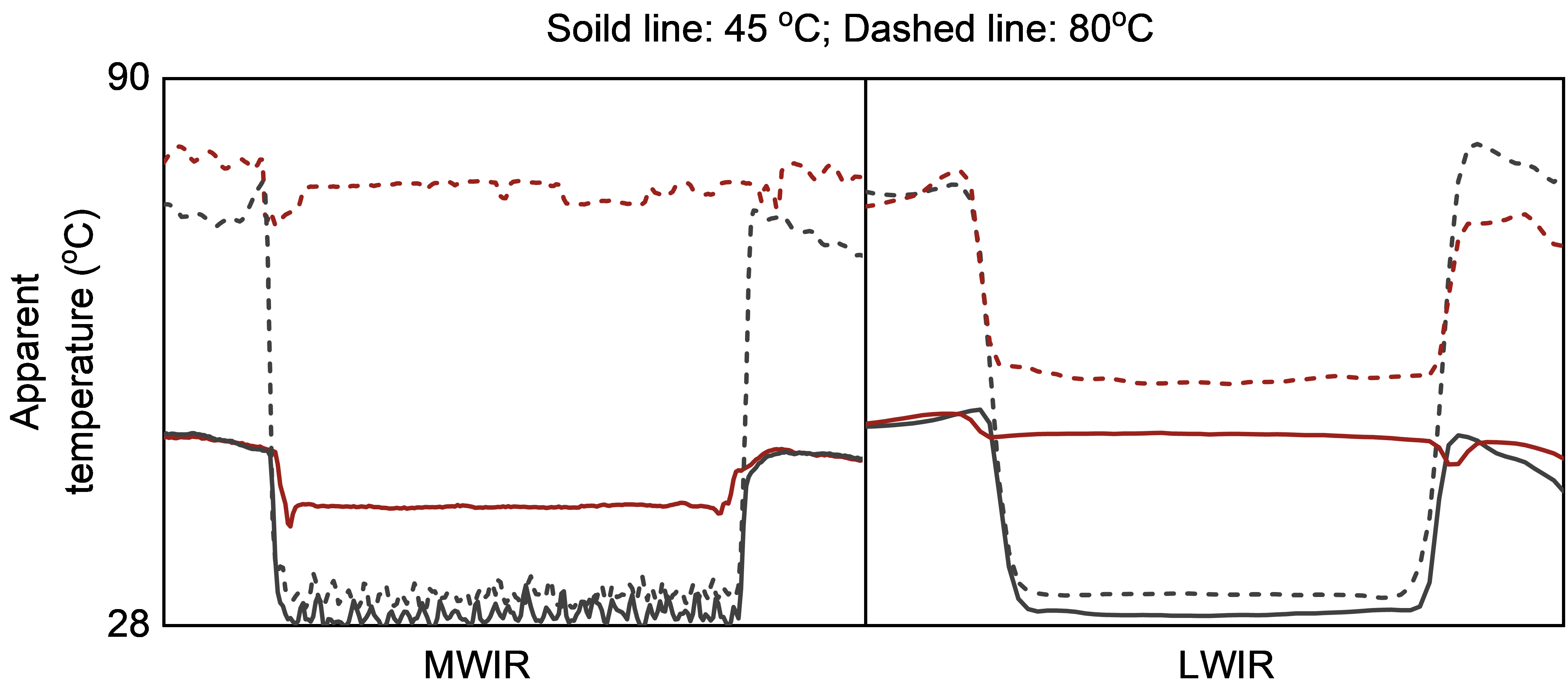


**Fig. S13** Line profiles of the apparent temperature across the cross-sectional area analyzed from the MWIR and LWIR thermal images in Fig. 4d.


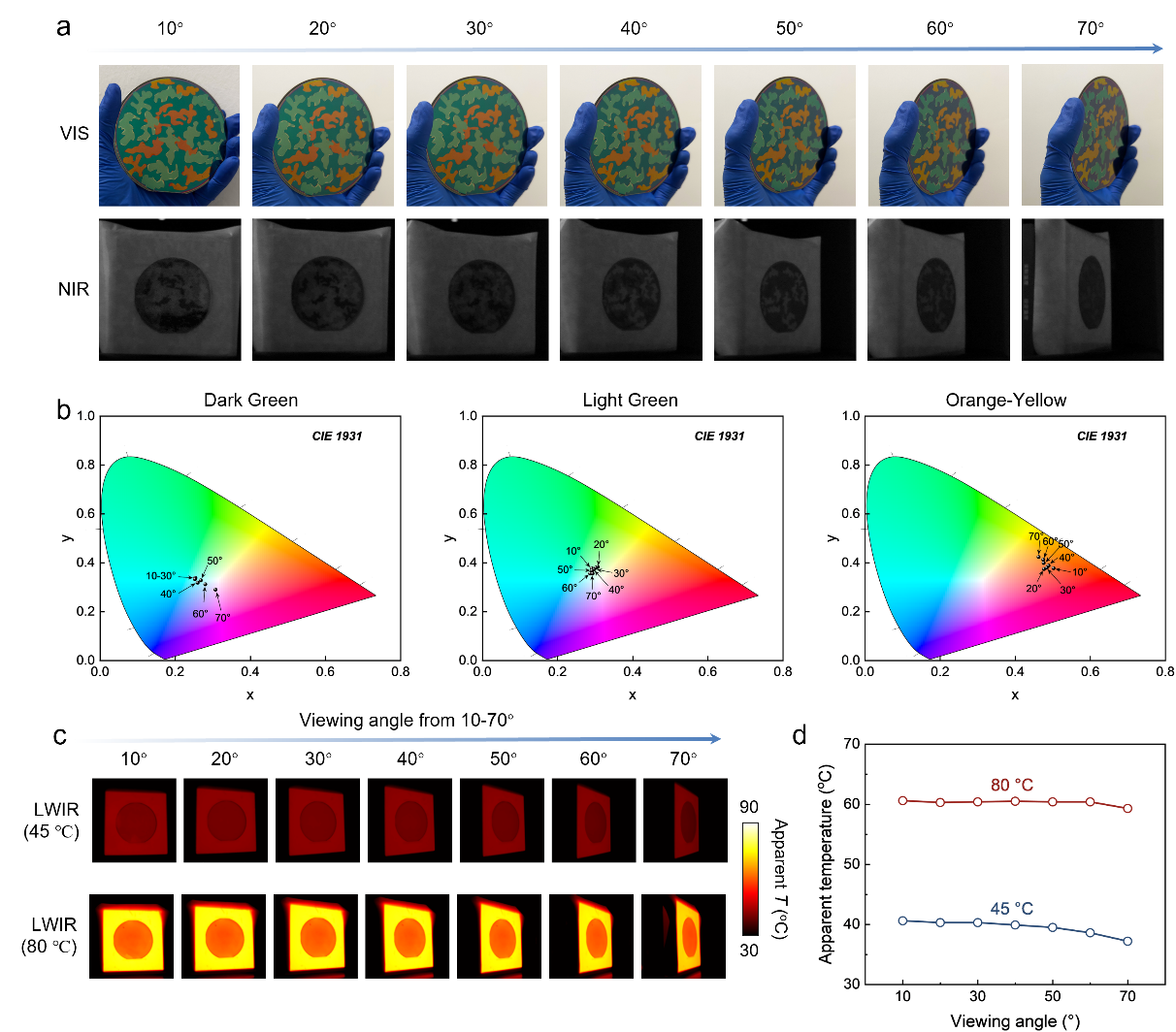


**Fig. S14** (a) Optical and NIR photographs of the patterned sample captured at different viewing angles. (b) Chromaticity coordinates of the three camouflage colors on the patterned sample under different viewing angles. (c) LWIR thermal images of the patterned sample captured at different viewing angles and temperatures. (d) Measured apparent temperatures of the sample surface as a function of viewing angle at different physical temperatures.


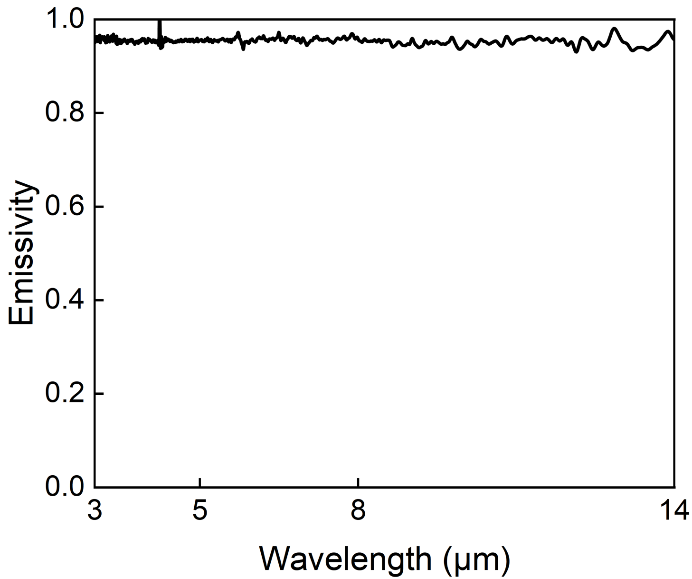


**Fig. S15** Measured emissivity spectrum of the background used in Figs. 5b and 5c.


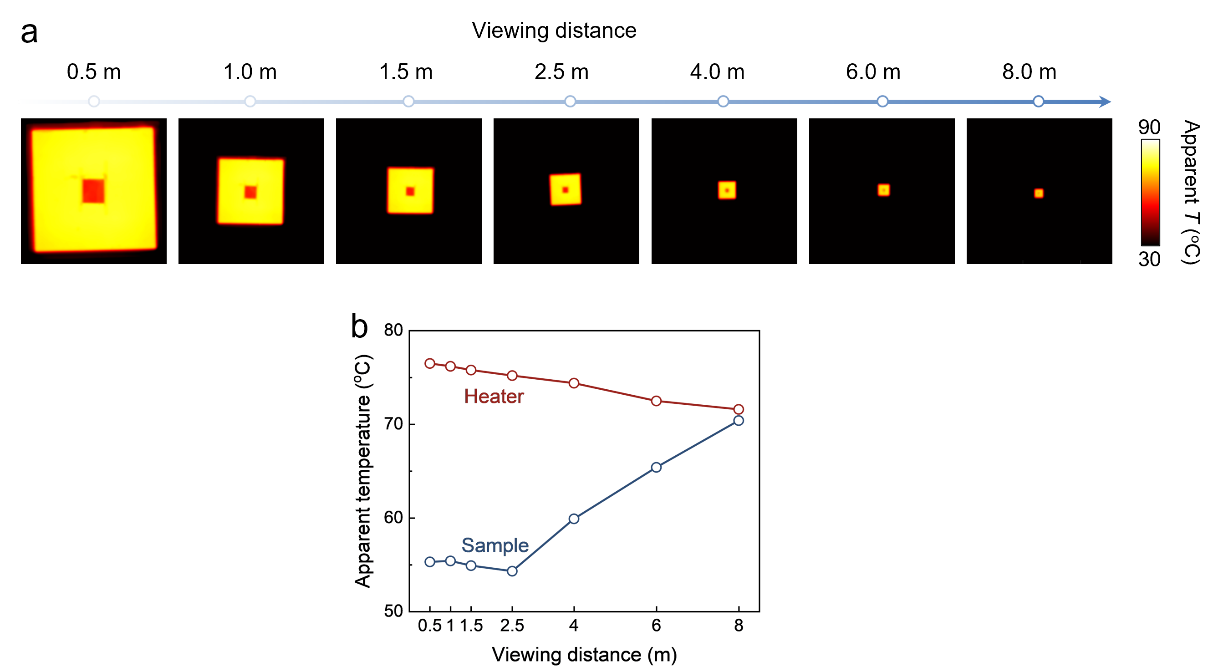


**Fig. S16 Determination of the reliable thermal decoding parameter space.** The effective decoding distance (*L*) based on geometric optics is calculated using the equation *L*=*d*_feature_/(*N*×IFOV), where dfeature represents the feature size and IFOV denotes the instantaneous field of view. Adopting the technical recommendation from FLIR (the MWIR/LWIR detector manufacturer) that a feature must cover at least 3×3 pixels for accurate radiometric measurement (*N*=3), and using our sample size of 3×3 cm along with the IFOVs of our MWIR (0.6 mrad) and LWIR (3.7 mrad) cameras, the theoretical reliable ranges are calculated as 0.3–16.6 m and 0.36–2.7 m, respectively. (a) LWIR thermal images of the 3×3 cm sample captured at different observation distances. (b) Measured apparent temperatures of the sample and the background (heating stage) at different distances.


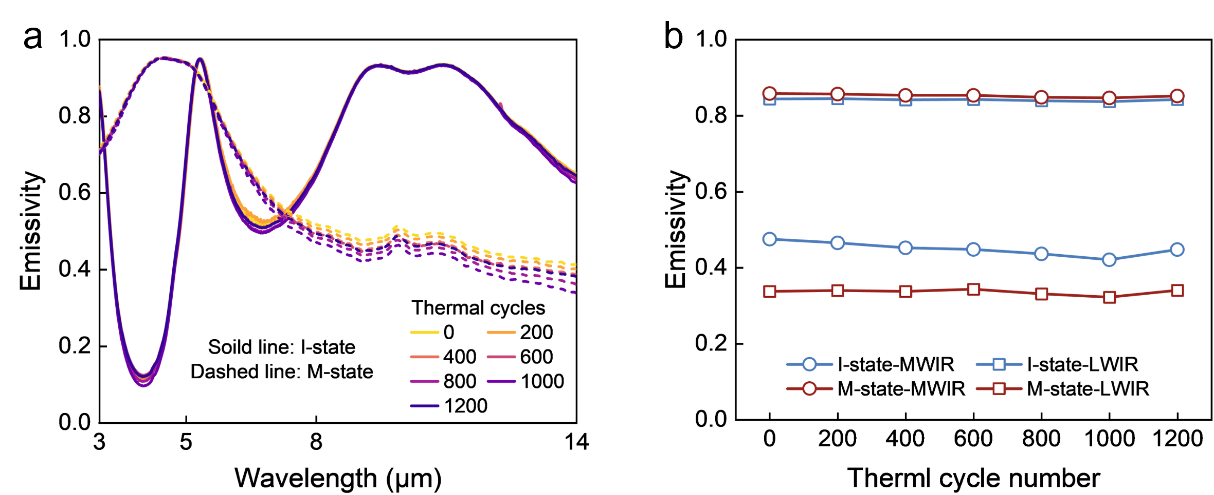


**Fig. S17** (a) Measured emissivity spectra of the sample (I- and M-state transition) after different numbers of thermal cycles. (b) Statistical average emissivity in MWIR and LWIR bands (I- and M-state transition) as a function of cycle number.


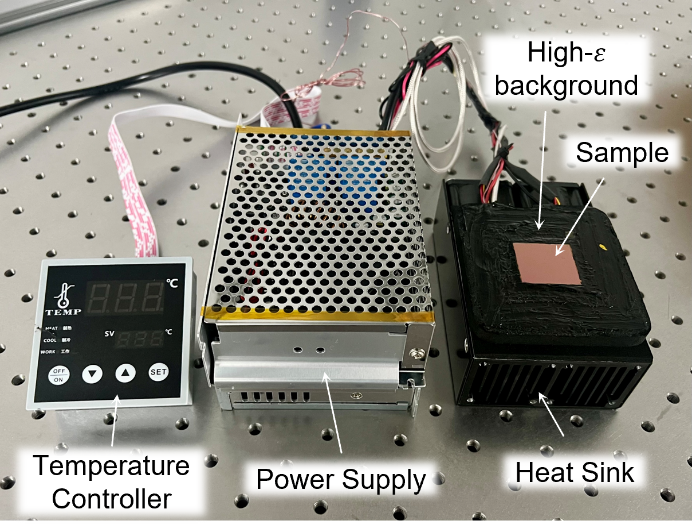


**Fig. S18** Optical photograph of the experimental setup for a single unit cell used to demonstrate dynamic data transmission.


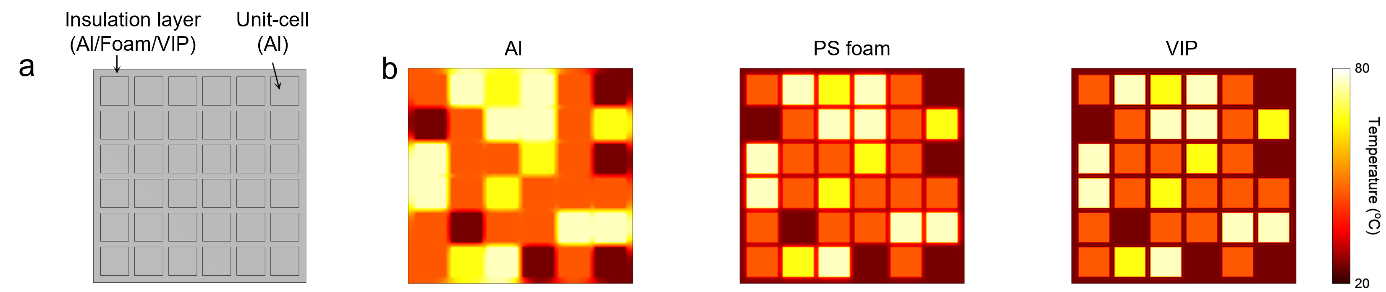


**Fig. S19** Numerical simulation of thermal crosstalk suppression in the arrayed device. To quantitatively verify the feasibility of crosstalk suppression, a 3D model was established using the Heat Transfer in Solids module in COMSOL Multiphysics. The model replicates the 6×6 array configuration (conceptualized in Figure 5e) with a unit pitch of 5×5 cm and a gap width of 0.5 cm. The active heating units were defined as Aluminum, and we considered natural convection and conduction under outdoor conditions with a surface non-radiative heat coefficient of 15 W m⁻² K⁻¹. The emissivity of all surfaces was set to 0.95 to account for radiative heat exchange with the ambient. The thermal transport properties were defined as follows: (1) Aluminum: Thermal conductivity *k* = 238 W/(m·K), Density *ρ* = 2700 kg/m³, Heat capacity at constant pressure *C*_p_ = 900 J/(kg·K); (2) Polystyrene (PS) Foam: *k* = 0.025 W/(m·K), *ρ* = 30 kg/m³, *C*_p_ = 1500 J/(kg·K); (3) Vacuum Insulation Panel (VIP): *k* = 0.004 W/(m·K), *ρ* = 200 kg/m³, *C*_p_ = 850 J/(kg·K). (a) Schematic of the simulation model for the arrayed device utilizing different gap-filling insulation materials. (b) Simulated steady-state temperature distributions with Aluminum (Al, left), Polystyrene Foam (middle), and Vacuum Insulation Panel (VIP, right) as the gap-filling material.


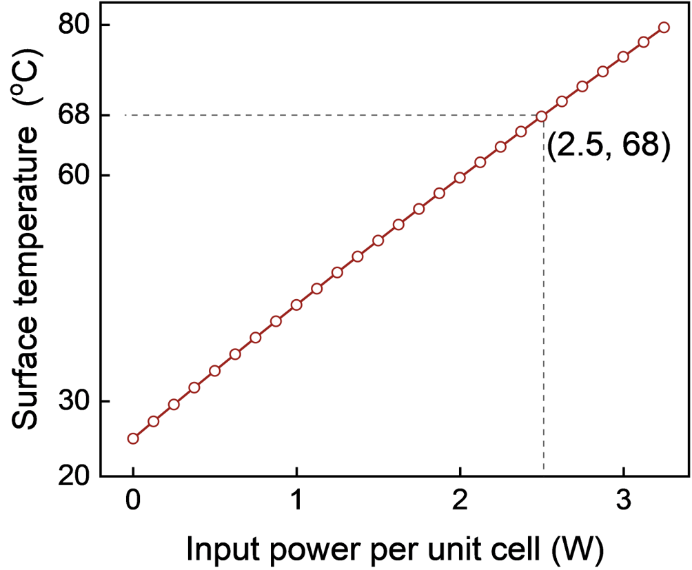


**Fig. S20 Energy Consumption Estimation.** To estimate the power budget, the average steady-state surface temperature distribution of the device was simulated using COMSOL Multiphysics by coupling the Heat Transfer in Solids and Surface-to-Surface Radiation. The model setup and boundary conditions were defined as follows: the active heating units were defined as Aluminum, and the gap-filling material was set as Vacuum Insulation Panel (VIP); the backside of the device was set as adiabatic, while the top surface exchanges heat with the ambient environment (25 ℃) through both thermal radiation (surface emissivity *ε*= 0.95) and natural convection and conduction, characterized by a non-radiative heat coefficient of 15 W m^-2^K^-1^. For the array configuration, the unit cells were assigned four distinct temperatures corresponding to the encoding states consistent with the conceptual diagram in Figure 5e. Figure S20 display the calculated steady-state surface temperature of a single 5×5 cm unit cell as a function of input electrical power. The results show a linear relationship, indicating that approximately 2.5 W per unit is required to reach the maximum encoding temperature of 68 ℃ (corresponding to logic signal "11").


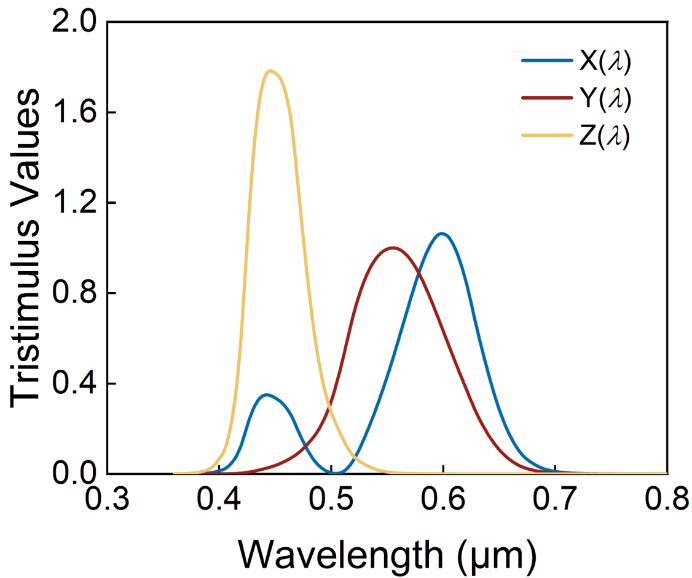


**Fig. S21** CIE color matching functions.

**Table S1** Thicknesses of the top three layers (TiO_2_/top Si/Ge) for the 15 photonic structure samples with different colors shown in Figure 3a. These are nominal thicknesses estimated from the deposition rate and time, rather than directly measured values.

|  | *h*_Ge_ (nm) | *h*_top Si_ (nm) | *h*_TiO2_ (nm) |
| --- | --- | --- | --- |
| #1-1 | 97 | 115 | 0 |
| #1-2 | 97 | 115 | 58 |
| #1-3 | 97 | 115 | 96 |
| #1-4 | 97 | 115 | 128 |
| #1-5 | 97 | 115 | 155 |
| #2-1 | 121 | 86 | 0 |
| #2-2 | 121 | 86 | 22 |
| #2-3 | 121 | 86 | 65 |
| #2-4 | 121 | 86 | 100 |
| #2-5 | 121 | 86 | 127 |
| #3-1 | 145 | 57 | 0 |
| #3-2 | 145 | 57 | 22 |
| #3-3 | 145 | 57 | 65 |
| #3-4 | 145 | 57 | 100 |
| #3-5 | 145 | 57 | 127 |

**Table S2** Thicknesses of the top three layers (TiO_2_/top Si/Ge) corresponding to the different color regions on the surface of the camouflage sample shown in Figure 4a. These are nominal thicknesses estimated from the deposition rate and time, rather than directly measured values.

|  | *h*_Ge_ (nm) | *h*_top Si_ (nm) | *h*_TiO2_ (nm) |
| --- | --- | --- | --- |
| Light Green | 97 | 115 | 54 |
| Orange-Yellow | 97 | 115 | 90 |
| Dark Green | 97 | 115 | 134 |

**Table S3** Thicknesses of the top three layers (TiO_2_/top Si/Ge) corresponding to the different color regions on the surface of the camouflage sample shown in Figure S10 (left). These are nominal thicknesses estimated from the deposition rate and time, rather than directly measured values.

|  | *h*_Ge_ (nm) | *h*_top Si_ (nm) | *h*_TiO2_ (nm) |
| --- | --- | --- | --- |
| Color 1 | 97 | 115 | 36 |
| Color 2 | 97 | 115 | 60 |
| Color 3 | 97 | 115 | 90 |

**Table S4** Thicknesses of the top three layers (TiO_2_/top Si/Ge) corresponding to the different color regions on the surface of the camouflage sample shown in Figure S10 (right). These are nominal thicknesses estimated from the deposition rate and time, rather than directly measured values.

|  | *h*_Ge_ (nm) | *h*_top Si_ (nm) | *h*_TiO2_ (nm) |
| --- | --- | --- | --- |
| Color 1 | 97 | 115 | 44 |
| Color 2 | 97 | 115 | 73 |
| Color 3 | 97 | 115 | 109 |

**Table S5** Detailed specifications of the optical imaging detectors (VIS, NIR, and SWIR).

| Band | Device | Detector Type | Resolution | Dynamic Range |
| --- | --- | --- | --- | --- |
| VIS | iPhone 13 Pro | CMOS | 4032×3024 | - |
| NIR | OV1080P | CMOS | 1920×1080 | 69 dB |
| SWIR | OWL-640S | Cooled InGaAs | 640×512 | 61 dB |

**Table S6** Detailed specifications of the thermal infrared detectors (MWIR and LWIR).

| Band | Device/ Detector Type | IFOV | Resolution | NETD | Integration Time/  Thermal Time Constant |
| --- | --- | --- | --- | --- | --- |
| MWIR | FLIR A6700  (Cooled InSb) | 0.6 mrad | 640×512 | < 25 mK | 1.28 ms  (Integration Time) |
| LWIR | FLIR E5 pro  (VOx) | 3.7 mrad | 160×120 | <60 mK | 7-12 ms  (Thermal Time Constant) |

**Table S7** Specifications of the active laser measurement system.

| Wavelength | Device | Detector Type | Resolution | Detection Metric |
| --- | --- | --- | --- | --- |
| Laser | FARO Focus S350 | Terrestrial  Laser Scanner | Up to  2MPts/sec | Laser High  Dynamic Range |

**Table S8** Magnetron sputtering parameters for each layer of the photonic structure.

| Layer | Power | Gas (sccm) | Pressure (Pa) |
| --- | --- | --- | --- |
| Mo | 50 W DC | Ar 45 | 0.4 |
| Si | 80 W RF | Ar 50 | 0.3 |
| ITO | 60 W RF | Ar 40 | 0.4 |
| VO_2_ | 200 W DC | Ar 45 / O_2_ 10 | 0.5 |
| Ge | 70 W RF | Ar 40 | 0.3 |
| TiO_2_ | 80 W RF | Ar 40 | 0.3 |
